# Supplementary material for: Subfield-specific interneuron circuits govern the hippocampal response to novelty in male mice
Source: Nat Commun. 2024 Jan 24;15:714. doi: 10.1038/s41467-024-44882-3 (PMC10808551; doi:10.1038/s41467-024-44882-3)
Supplement: Supplementary file 1 — Supplementary Information [file 41467_2024_44882_MOESM1_ESM.pdf]

Subfield-specific interneuron circuits govern the hippocampal response to novelty in male mice

Thomas Hainmueller, Aurore Cazala, Li-Wen Huang, and Marlene Bartos

Supplementary Materials

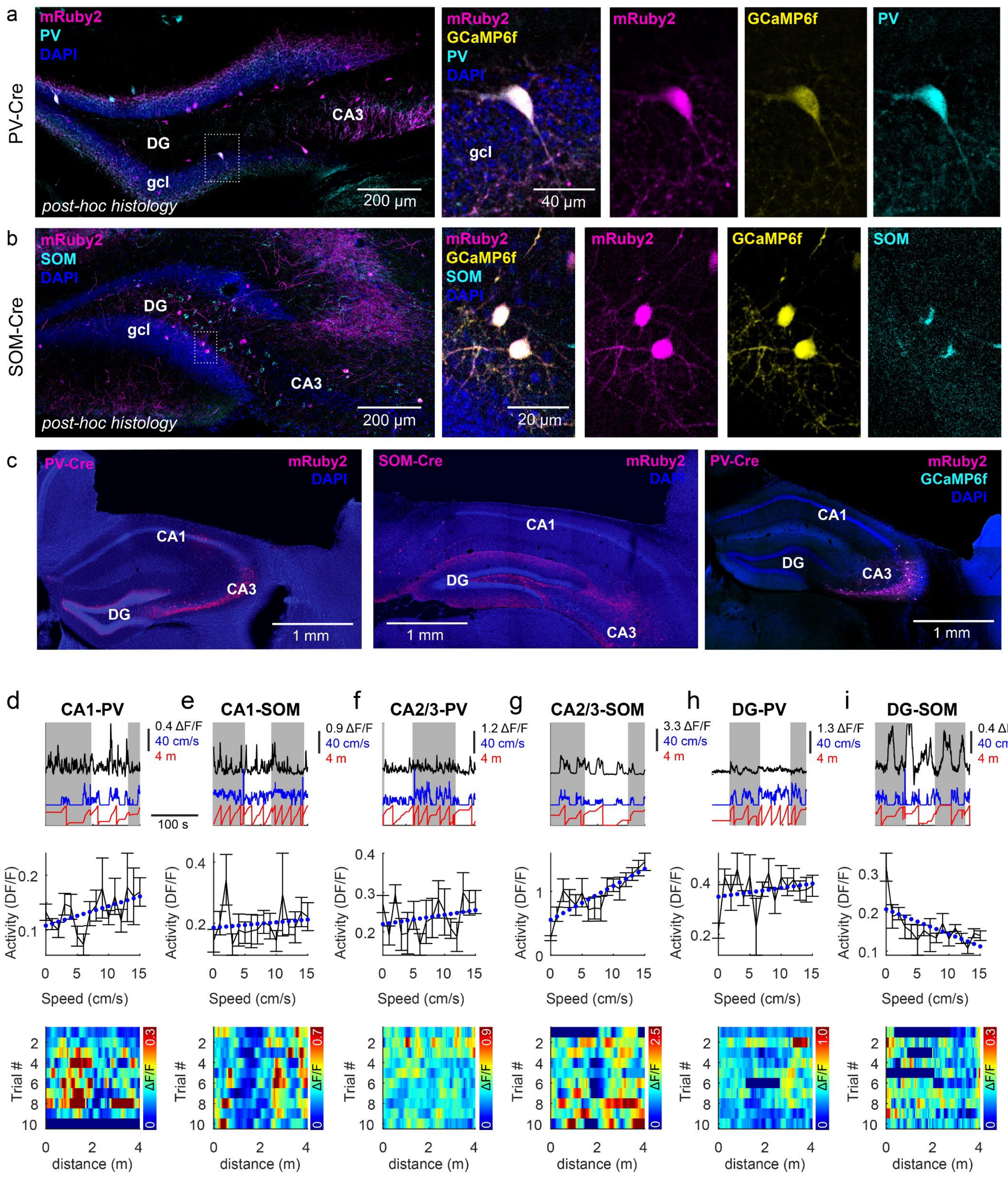

a
PV-Cre
mRuby2
PV
DAPI
DG
gcl
CA3
post-hoc histology
200 µm
mRuby2
GCaMP6f
PV
DAPI
gcl
40 µm
b
SOM-Cre
mRuby2
SOM
DAPI
DG
gcl
CA3
post-hoc histology
200 µm
20 µm
c
PV-Cre
SOM-Cre
mRuby2
GCaMP6f
DAPI
CA1
CA3
DG
1 mm
d CA1-PV
e CA1-SOM
f CA2/3-PV
g CA2/3-SOM
h DG-PV
i DG-SOM
0.4 ΔF/F
0.9 ΔF/F
1.2 ΔF/F
3.3 ΔF/F
1.3 ΔF/F
40 cm/s
4 m
100 s
Activity (DF/F)
Speed (cm/s)
Trial #
ΔF/F
distance (m)

Supplementary Figure 1 | Cell examples. a Post-hoc confocal fluorescence image of a brain slice from an implanted PV-Cre animal. Fluorescence of mRuby2 (*magenta*), GCaMP6f (*yellow*), PV immunolabelling (*cyan*) and DAPI (*blue*) is shown. Dashed boxes in the left image denote the location of the magnified inset on the right. Abbreviations: DG dentate gyrus; gcl granule cell layer. Section is representative for n = 3 animals in which confirmatory immunohistochemistry was performed. **b** Same as in a, but for a SOM-Cre animal. Section is representative for n = 3 animals in which confirmatory immunohistochemistry was performed. **c** Sections from three implanted animals at low magnification. *Left,* PV-Cre animal, *middle* SOM-Cre animal with dorsal window implantation for DG and CA1 imaging, *right* shows a PV-Cre mouse implanted with a lateral window for CA3 recordings. Similar histology was obtained for n = 15 mice used for interneuron imaging experiments. **d-i** Each column shows examples of raw recording data (*top*), speed modulation (*middle*) and spatial distribution of activity (*bottom*) for one neuron from each region and interneuron (IN) class. Top row, traces on top show IN activity (*black*), animal movement running speed (*blue*) and location on the virtual track (*red*). Middle row shows mean ± SEM of activity as a function of running speed, blue dotted lines represent the linear fit to these data. Bottom row shows color-coded activity over track distance (x-axis) for multiple runs (y-axis) on the familiar track.

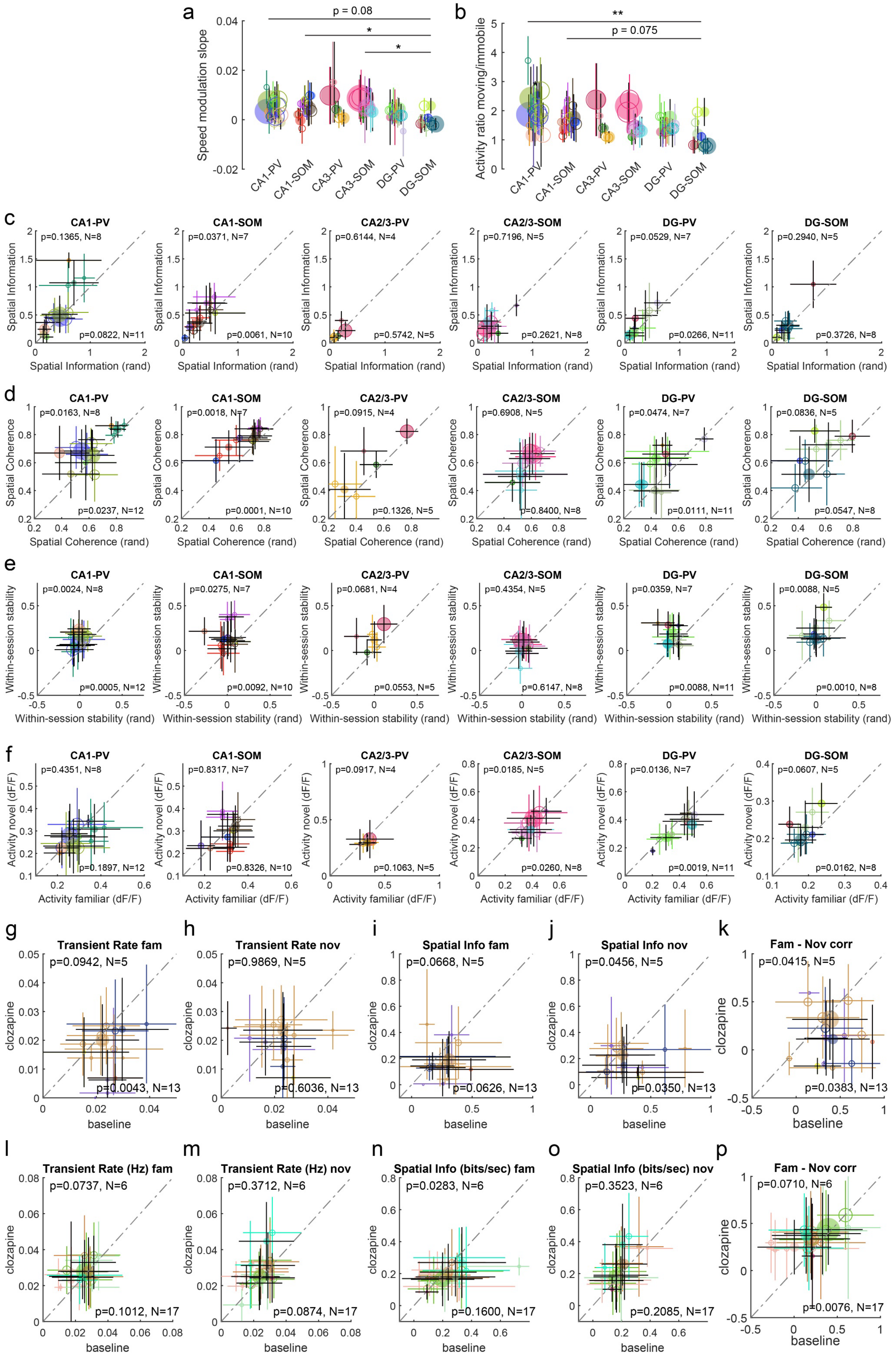

a
Speech modulation slope
p = 0.08
*
*
CA1-PV
CA1-SOM
CA3-PV
CA3-SOM
DG-PV
DG-SOM
b
Activity ratio moving/immobile
**
p = 0.075
c
Spatial Information
Spatial Information (rand)
CA1-PV p=0.1365, N=8 p=0.0822, N=11
CA1-SOM p=0.0371, N=7 p=0.0061, N=10
CA2/3-PV p=0.6144, N=4 p=0.5742, N=5
CA2/3-SOM p=0.7196, N=5 p=0.2621, N=8
DG-PV p=0.0529, N=7 p=0.0266, N=11
DG-SOM p=0.2940, N=5 p=0.3726, N=8
d
Spatial Coherence
Spatial Coherence (rand)
CA1-PV p=0.0163, N=8 p=0.0237, N=12
CA1-SOM p=0.0018, N=7 p=0.0001, N=10
CA2/3-PV p=0.0915, N=4 p=0.1326, N=5
CA2/3-SOM p=0.6908, N=5 p=0.8400, N=8
DG-PV p=0.0474, N=7 p=0.0111, N=11
DG-SOM p=0.0836, N=5 p=0.0547, N=8
e
Within-session stability
Within-session stability (rand)
CA1-PV p=0.0024, N=8 p=0.0005, N=12
CA1-SOM p=0.0275, N=7 p=0.0092, N=10
CA2/3-PV p=0.0681, N=4 p=0.0553, N=5
CA2/3-SOM p=0.4354, N=5 p=0.6147, N=8
DG-PV p=0.0359, N=7 p=0.0088, N=11
DG-SOM p=0.0088, N=5 p=0.0010, N=8
f
Activity novel (dF/F)
Activity familiar (dF/F)
CA1-PV p=0.4351, N=8 p=0.1897, N=12
CA1-SOM p=0.8317, N=7 p=0.8326, N=10
CA2/3-PV p=0.0917, N=4 p=0.1063, N=5
CA2/3-SOM p=0.0185, N=5 p=0.0260, N=8
DG-PV p=0.0136, N=7 p=0.0019, N=11
DG-SOM p=0.0607, N=5 p=0.0162, N=8
g Transient Rate fam p=0.0942, N=5 p=0.0043, N=13
h Transient Rate nov p=0.9869, N=5 p=0.6036, N=13
i Spatial Info fam p=0.0668, N=5 p=0.0626, N=13
j Spatial Info nov p=0.0456, N=5 p=0.0350, N=13
k Fam - Nov corr p=0.0415, N=5 p=0.0383, N=13
l Transient Rate (Hz) fam p=0.0737, N=6 p=0.1012, N=17
m Transient Rate (Hz) nov p=0.3712, N=6 p=0.0874, N=17
n Spatial Info (bits/sec) fam p=0.0283, N=6 p=0.1600, N=17
o Spatial Info (bits/sec) nov p=0.3523, N=6 p=0.2085, N=17
p Fam - Nov corr p=0.0710, N=6 p=0.0076, N=17
clozapine
baseline

Supplementary Figure 2 | Data organized by animal and session ID. **a** Speed-modulation slope organized by animals and sessions (related to **Fig. 2c**). *Filled circles* with black lines indicate per-animal means and standard deviations. *Open circles* with colored lines indicate per-session means and standard deviations. The size of the circle is proportional to the number of cells recorded in a given animal or session. See **Supplementary Table 1** for a tabulated summary of the numbers of cells and sessions recorded in each animal. A unique color was chosen to identify each animal, and this color code is applied consistently to the means from this animal and the means of all sessions recorded from this animal throughout all subpanels of this figure. *P* values in a,b indicate comparisons between the unweighted aggregate means of recording sessions (see **Supplementary Table 2** for a full set of comparisons between all groups and for data aggregated by animal). **b** Same as in a but for activity ratios between running or in the absence of directed movement (immobile). Related to **Fig. 2d**. **c** Spatial information against chance levels averaged for sessions (*open circles*) and animals (*filled circles*; related to **Fig. 3c**). Remarks to a apply here, too. Top row *p* and N values in c-p indicate statistical comparisons on averaged values of data aggregated by animal, bottom row statistics for averages of data aggregated by recording session. **d** Same as in c but for spatial coherence (related to **Fig. 3d**). **e** Same as in c but for within-session stability (related to **Fig. 3e**). **f** Mean activity during movement in the familiar (*x-axis*) and novel (*y-axis*) context averaged over animals (*filled circles*) and recording sessions (*open circles*). Remarks to a and c apply here, too (related to **Fig. 4a,b**). **g** Average calcium transient rate of GCs with place fields on the familiar track in baseline (*x-axis*) and clozapine (*y-axis*) conditions in PV-Cre animals transfected with h4MDi, aggregated per animal (*filled circles*), or recording sessions (*open circles*). Remarks to a and c apply here, too (related to **Fig. 6c,** *left group*). **h** Same as in g but for calcium transient rates in the novel environment (related to **Fig. 6c,** *right group*). **i** Same as in g but for spatial information in the familiar environment (related to **Fig. 6d,** *left group*). **j** Same as in g but for spatial information in the novel environment (related to **Fig. 6d,** *right group*). **k** Correlations of spatial activity profiles ('place fields') between familiar and novel environments before (baseline) and after (clozapine) suppression of DG PV-IN activity *via* h4MDi. *Filled circles* show averages of data aggregated by animal, *open circles* averages of data aggregated by recording session. Remarks to a and c apply here, too (related to **Fig. 7b**). **l** Same as in g but for SOM-Cre animals transfected with h4MDi (related to **Fig 6g,** *left group*). **m** Same as in h but for SOM-Cre animals transfected with h4MDi (related to **Fig. 6g,** *right group*). **n** Same as in i but for SOM-Cre animals transfected with h4MDi (related to **Fig 6h,** *left group*). **o** Same as in j but for SOM-Cre animals transfected with h4MDi (related to **Fig. 6h,** *right group*). **p** Same as in k but for SOM-Cre animals transfected with h4MDi (related to **Fig. 7d**). a,b Kruskal-Wallis ANOVA on ranks with Dunn's post-hoc test c-p Paired t-test. *$p < 0.05$; **$p < 0.01$, For exact *p* values see **Supplementary Table 2**.

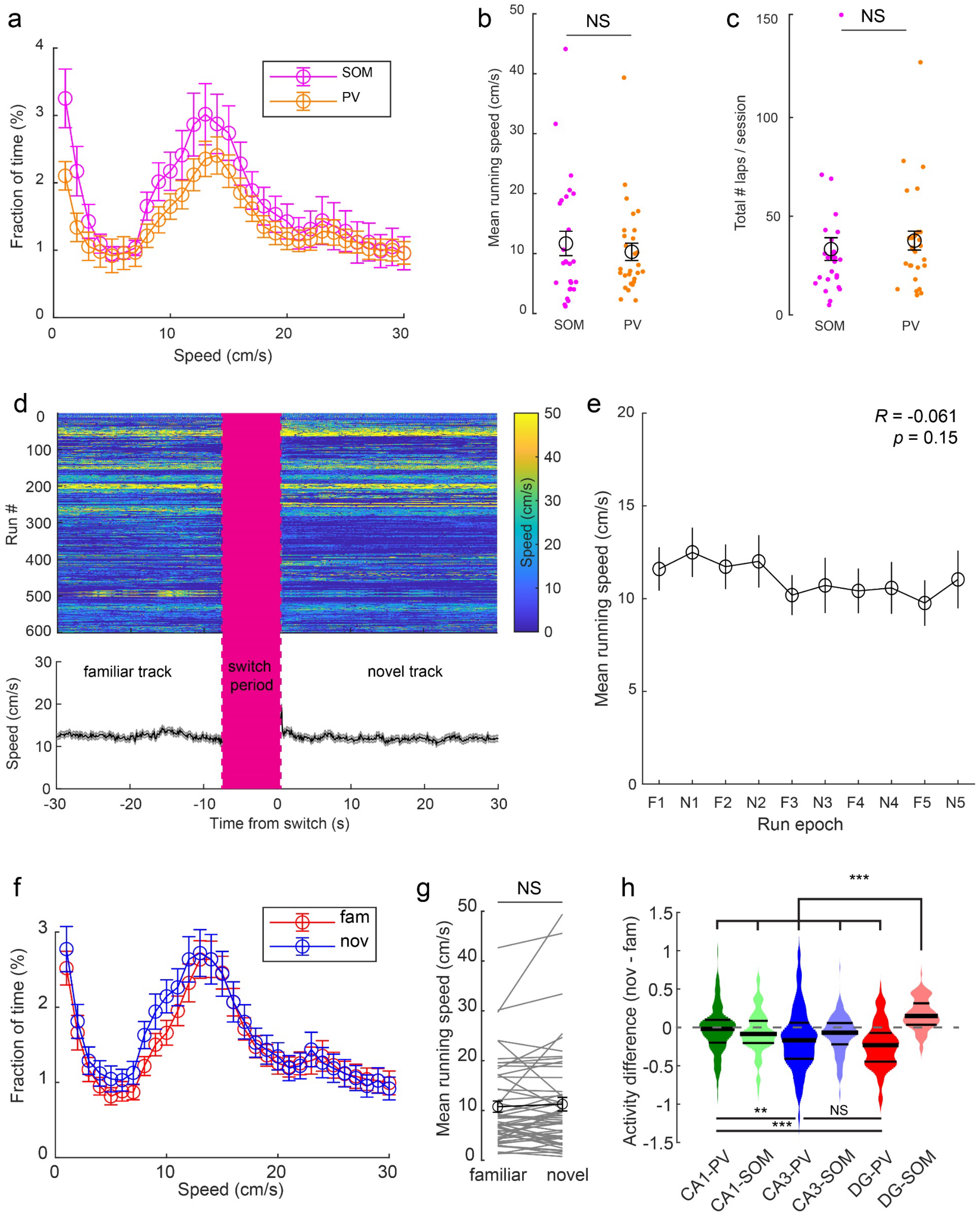
a
SOM
PV
Fraction of time (%)
Speed (cm/s)
b
NS
Mean running speed (cm/s)
SOM
PV
c
NS
Total # laps / session
SOM
PV
d
Run #
Speed (cm/s)
familiar track
switch period
novel track
Speed (cm/s)
Time from switch (s)
e
R = -0.061
p = 0.15
Mean running speed (cm/s)
F1
N1
F2
N2
F3
N3
F4
N4
F5
N5
Run epoch
f
fam
nov
Fraction of time (%)
Speed (cm/s)
g
NS
Mean running speed (cm/s)
familiar
novel
h
***
Activity difference (nov - fam)
**
NS
***
CA1-PV
CA1-SOM
CA3-PV
CA3-SOM
DG-PV
DG-SOM

Supplementary Figure 3 | Familiar and novel context activity and behavior. a Distribution of running speeds for PV-Cre mice (*orange*) and SOM-Cre mice (*magenta*) averaged over familiar and novel context. Circles with bars denote mean ± SEM. Note speed profiles are similar between PV-Cre and SOM-Cre mice. **b** Mean running speed for each recording session in the SOM-Cre (*magenta*) and PV-Cre (*orange*) cohort. Circles with bars denote mean ± SEM. Student's t-test. **c** Total number of laps run on the circular track for each recording session in the SOM-Cre (*magenta*) and PV-Cre (*orange*) cohort. Circles with bars denote mean ± SEM. Wilcoxon Rank-Sum test. **d** Running-speed (color-coded) at familiar to novel context transition (*pink bar*) for all individually recorded runs (rows in *top plot*) and mean ± SEM (black line with shading, *bottom plot*). Note, running speed was not acquired during the switch period due to technical limitations. Mean running speed before and after the switch period was relatively constant (N = 32 mice). **e** Mean running speed ± SEM (circles with bars) for all subsequent circular track runs in the recording session averaged across recordings (N = 54 sessions from 32 mice). *X-axis* denotes sessions arranged by their timing, x-labels indicated familiar (F1,2,3,…) and novel (N1,2,3,…) track runs in their temporal order. Note, no significant decrease of mean running speeds over the course of behavioral sessions. **f** Distribution of running speeds in the familiar (*red*) and novel (*blue*) context for all recorded mice. Circles with bars denote mean ± SEM. **g** Comparison of mean running speed between familiar- and novel context runs for each session (*grey lines*). Black circles with bars denote mean ± SEM for all session. Paired t-test. **h** Distribution of activity differences obtained by subtracting mean z-scored activation on the familiar track from that on the novel one. Lines denote median and interquartile range. Kruskal-Wallis with Dunn's post-hoc test. NS not significant $p \geq 0.05$; **$p < 0.01$; ***$p < 0.0001$. See **Supplementary Table 2** for exact p values.

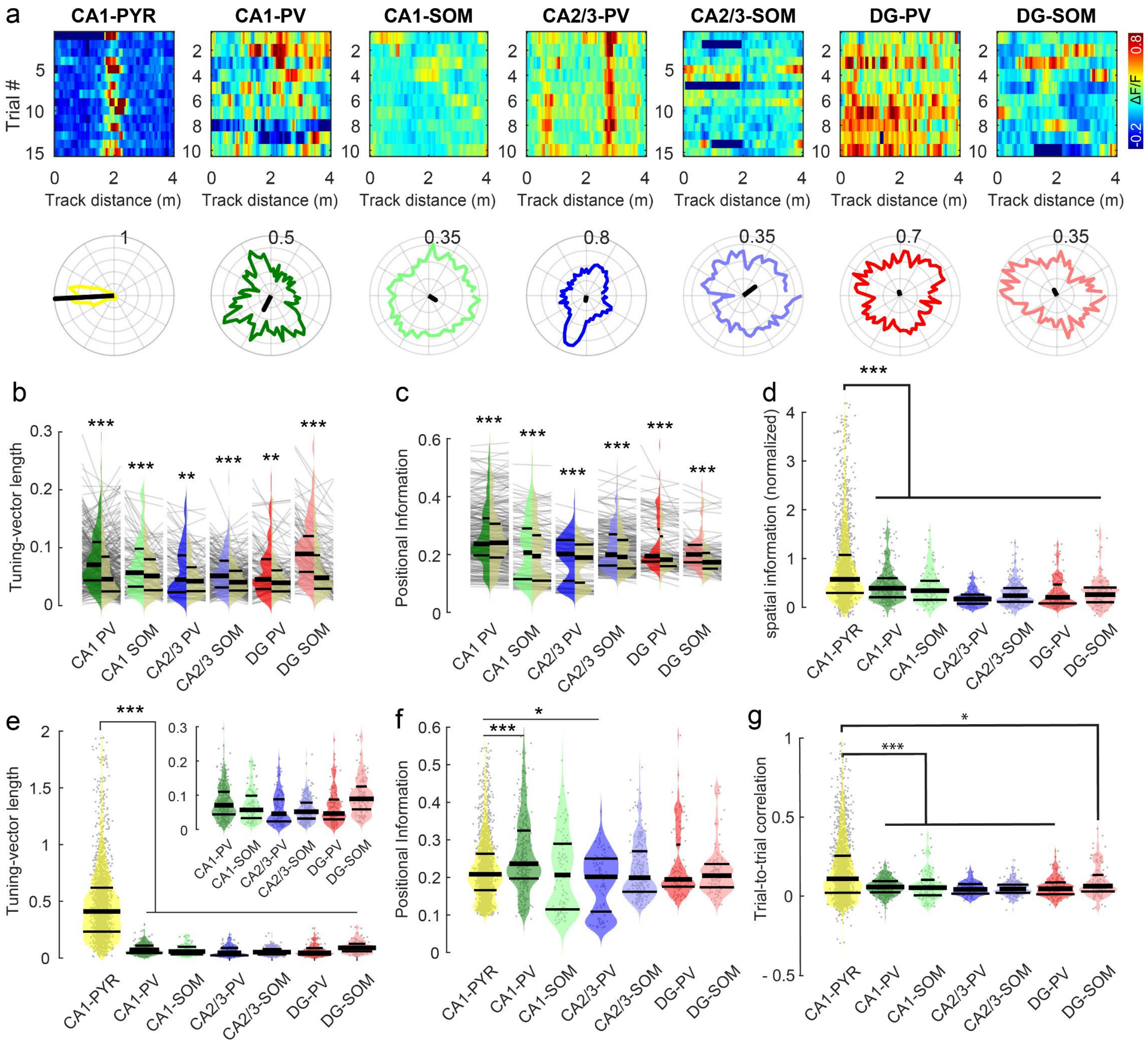

Supplementary Figure 4 | Comparison of spatial tuning parameters. a Examples of circular tuning analysis. Top row shows neuron activity (color-coded) over track location for multiple runs. Bottom row shows the circular tuning profiles of the mean spatial activity for the respective neurons. The mean tuning vector (see **Methods**) is shown in black. Concentric rings indicate activity levels (ΔF/F), numbers refer to the activity level of the outermost ring. **b** Distribution of tuning vector lengths (*left half of violin plots, colored*) vs. shuffle values (*right half of violin plots, beige*). **c** Same as in b but for positional information (see **Methods**). **d** Comparison of activity-normalized spatial information (SI) between CA1 principal cells (*yellow*) and INs in the various hippocampal subfields. **e** Same as in c but for tuning-vector length. Inset shows the distribution for INs on an expanded y-axis. **f** Same as in c, but for positional information. **g** Same as in c, but for trial-by-trial correlation of the spatial activity profile. Pyramidal cell activity is more self-similar across trials than that of hippocampal INs. b,c Wilcoxon signed rank-sum test. d-g Kruskal-Wallis with Dunn's post-hoc test. **$p < 0.01$; ***$p < 0.0001$. Lines represent median and interquartile ranges. Dots denote individual cells. See **Supplementary Table 2** for statistical comparisons between all groups and exact p values. The principal cell data in a and d-g are from recordings for Hainmueller and Bartos[10].

All cells

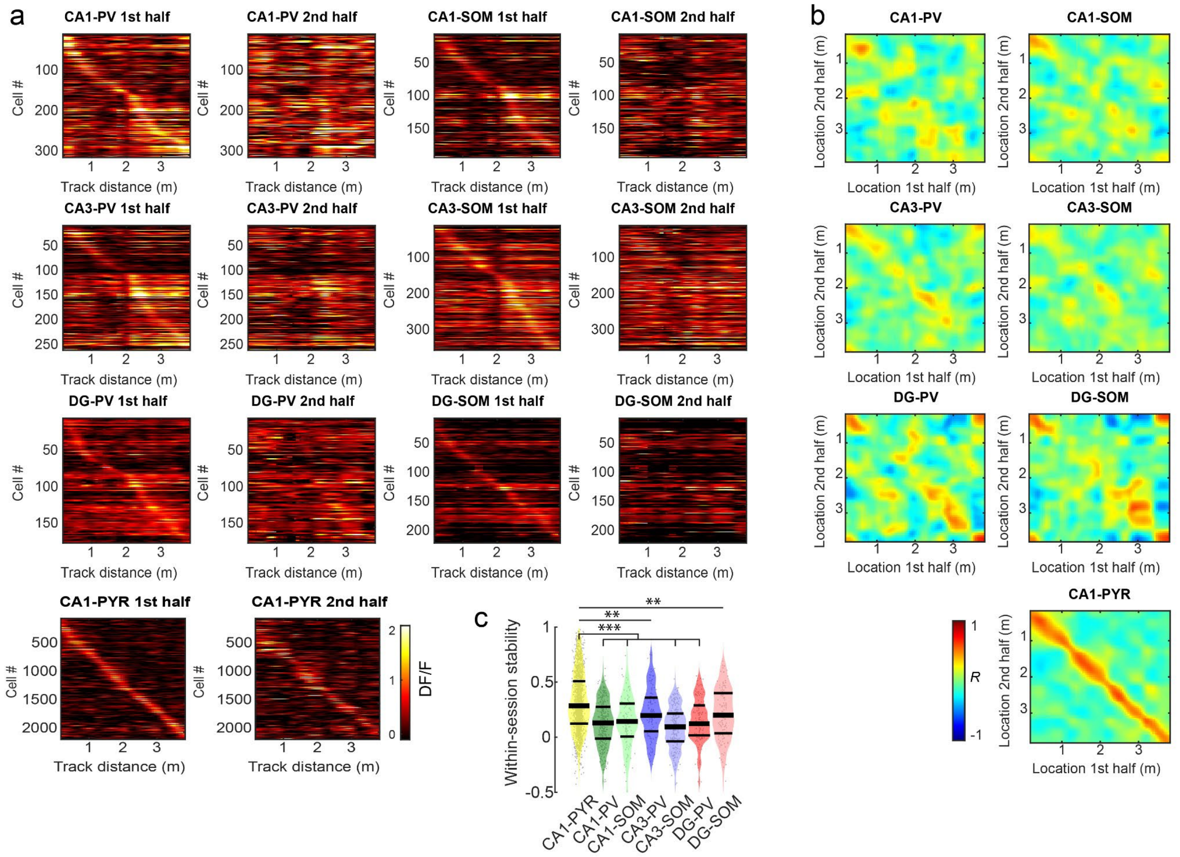

Cells with significant spatial information

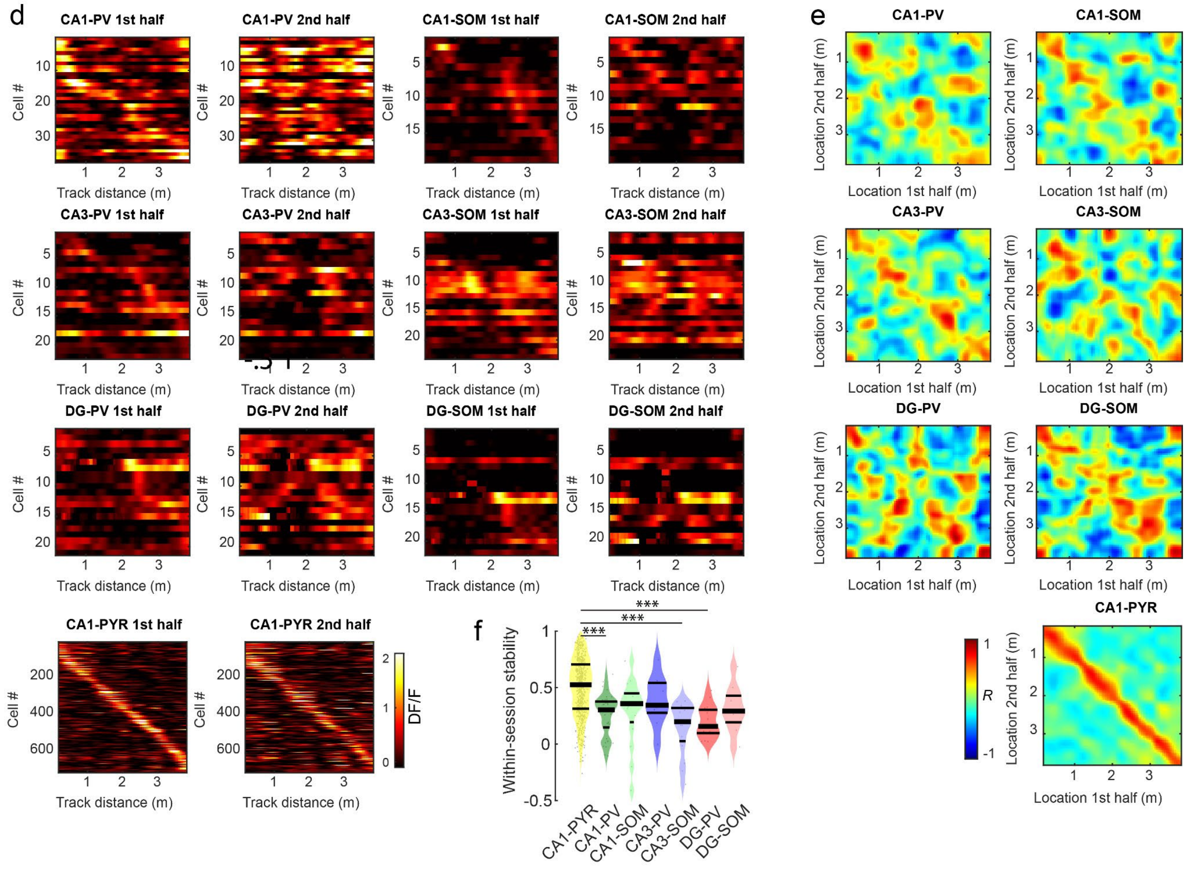

Supplementary Figure 5 | Within-session stability of spatial activity. a Activity (ΔF/F) over track distance. Cells were sorted according to their peak activity in the first half of familiar-track runs in one session, and are plotted separately for the first (*left*) and second (*right*) half of runs with that same sorting. **b** Correlation of population vectors between the first- and second half of runs on the familiar track as a function of track position (see **Methods**). Note, pyramidal cell population vectors (bottom row) show high correlation values along the identity line, indicating stable spatial activity patterns throughout the recording session. In comparison, these correlations are less pronounced in INs. **c** Mean correlation of spatial activity maps between the first- and second half of runs on the familiar track ('within-session stability') for all cell groups. **d-f** Same as a-c but only for preselected cells with significant spatial information. c,f Kruskal-Wallis with Dunn's post-hoc test. $^*p < 0.05$ $^{**}p < 0.01$; $^{***}p < 0.0001$. Lines represent median and interquartile ranges. See **Supplementary Table 2** for all statistical comparisons between groups and exact *p* values. The principal cell data in a and d-g are from recordings for Hainmueller and Bartos[10].

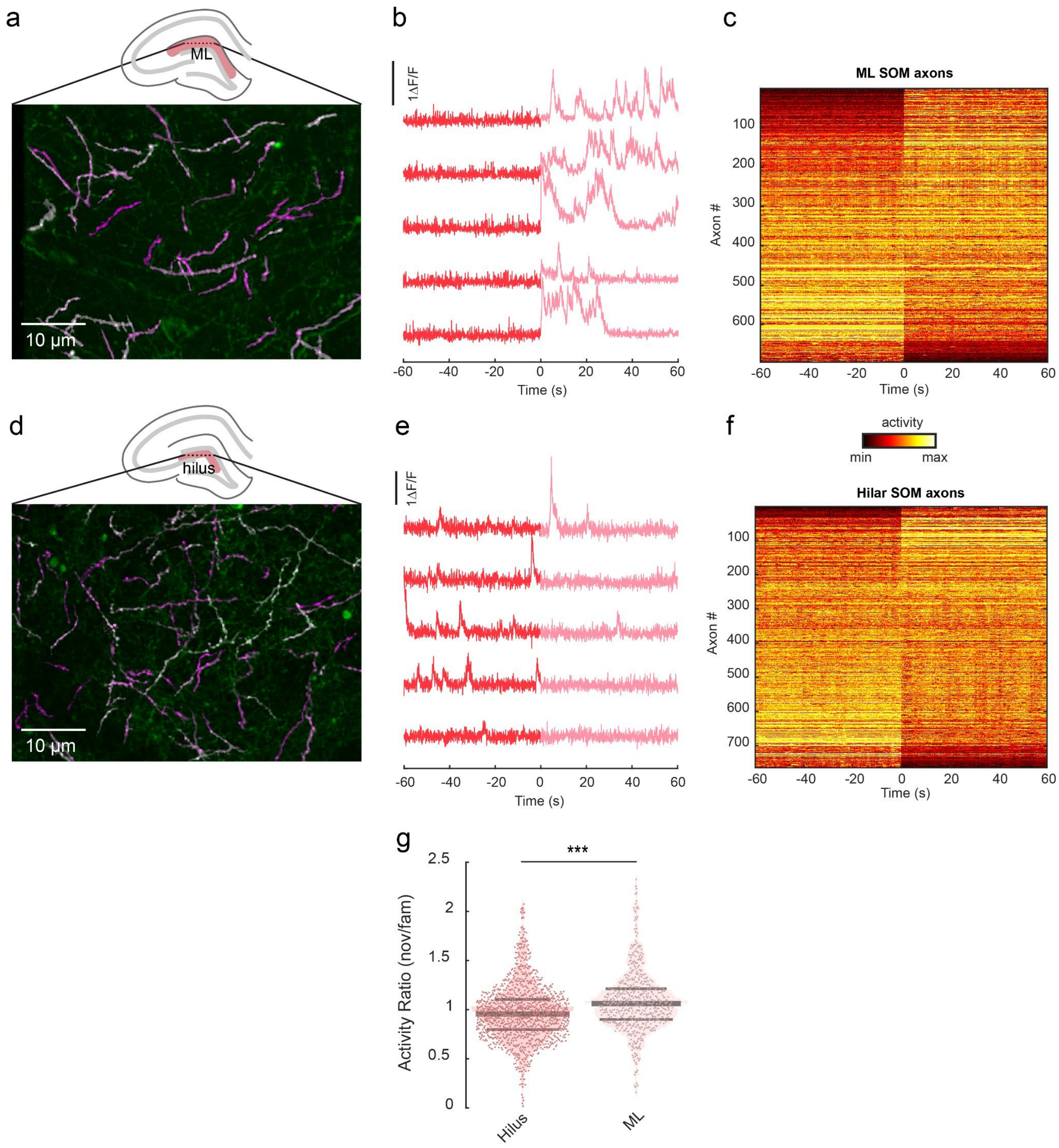

Supplementary Figure 6 | Recording of SOM axons in the DG. a Average GCaMP6f fluorescence (*green*) with highlighted ROIs (*magenta*) of SOM-IN axons in the DG molecular layer (ML). Illustrative for the recording conditions in n = 8 experiments. **b** Illustrative calcium traces from one SOM-IN axon in the DG ML aligned to the transition from the familiar to the novel environment at time point zero. Illustrative for the recording conditions in n = 8 experiments. **c** Mean calcium activity traces (normalized) aligned to the familiar-to-novel context transition for all ML SOM-IN axons recorded. Axons were sorted by their ratio of familiar divided by novel context activity. **d-f** Same as a-c but for SOM axons recorded in the DG hilus. **g** Ratios between calcium activity during running in familiar and novel environments for SOM-IN boutons in the DG hilus (*left*) and molecular layer (ML, *right*). Wilcoxon rank-sum test. *** $p < 0.001$, see **Supplementary Table 2** for exact *p* values.

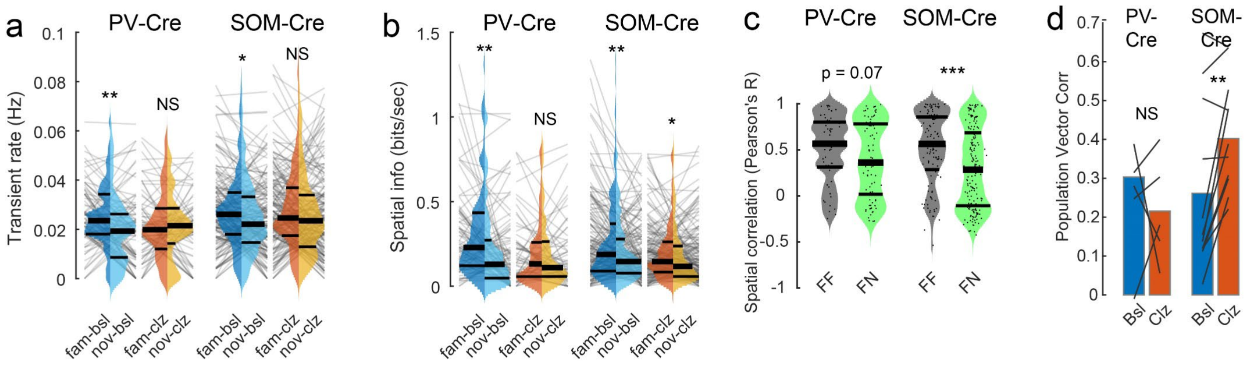

Supplementary Figure 7 | Chemogenetic manipulation of SOM and PV interneurons in the DG. a Average calcium-transient rate of GCs with place fields under control conditions (*blue group*) and under clozapine conditions (*yellow group*) in PV-Cre (*left ensemble*) and SOM-Cre mice (*right ensemble*) with h4MDi expression in the respective DG IN types. Note, significant drop of GC activity from the familiar to the novel context, which is absent after inhibition of PV- and SOM-INs. **b** Same as in a but for spatial information. Wilcoxon signed rank-sum test. **c** Place field correlation for GCs between one half of runs on the familiar context vs. the other half (FF, *grey*) and between runs on the familiar track vs. the novel track (FN, *green*) in PV-Cre (*left group*) and SOM-Cre (*right group*) under baseline conditions before injection of clozapine. Wilcoxon rank-sum test. **d** Population vector correlations between the familiar and novel context (see **Methods**) for sessions with at least 5 GC place cells during baseline (bsl, *blue*) and clozapine (clz, *orange*) conditions, respectively. *Left ensemble* shows data from PV-Cre animals injected with h4MDi, *right ensemble* shows the same for SOM-Cre animals. Paired t-test. *$p < 0.05$; **$p < 0.01$, ***$p < 0.001$. For exact p values see **Supplementary Table 2**. Lines on violin plots indicated median and interquartile ranges, respectively. Lines or dots denote values for individual cells.

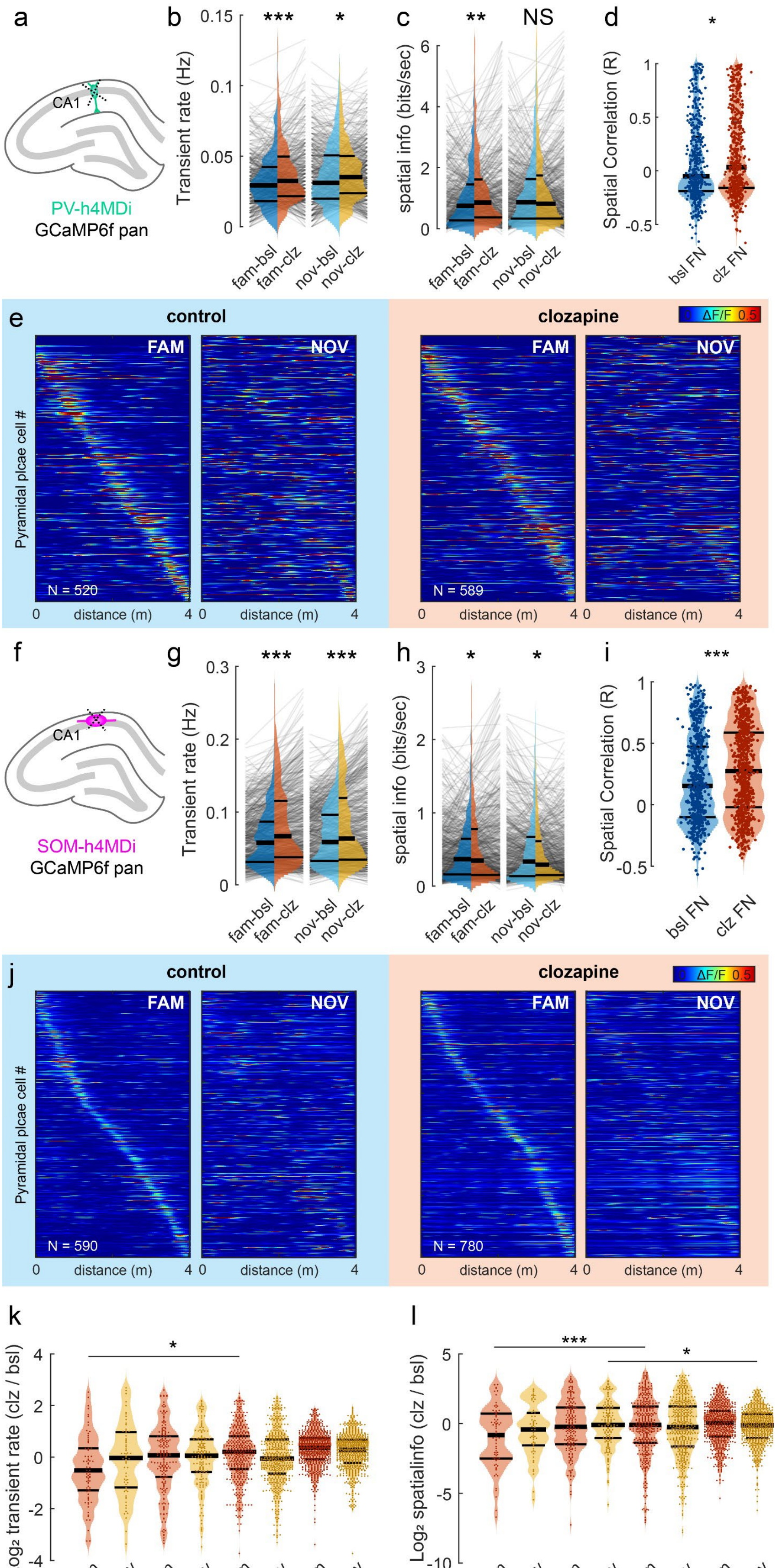

a
CA1
PV-h4MDi
GCaMP6f pan
b
Transient rate (Hz)
***
*
fam-bsl
fam-clz
nov-bsl
nov-clz
c
spatial info (bits/sec)
**
NS
d
Spatial Correlation (R)
*
bsl FN
clz FN
e
control
clozapine
ΔF/F 0.5
FAM
NOV
Pyramidal plcae cell #
N = 520
N = 589
distance (m)
f
SOM-h4MDi
GCaMP6f pan
g
h
i
***
j
N = 590
N = 780
k
Log₂ transient rate (clz / bsl)
DG PV Fam
DG PV Nov
DG SOM Fam
DG SOM Nov
CA1 PV Fam
CA1 PV Nov
CA1 SOM Fam
CA1 SOM Nov
l
Log₂ spatialinfo (clz / bsl)

Supplementary Figure 8 | Chemogenetic manipulation of SOM and PV interneurons in CA1. a Schematic illustration of the experiment: CA1 PV-IN were transfected with h4MDi inhibitory receptors to reversibly silence them by injecting clozapine intraperitoneally. **b** Average calcium-transient rate of CA1 pyramidal cells with place fields on the familiar track (*left group*) or novel track (*right group*) in baseline (bsl) and clozapine (clz) conditions. Thick black lines denote mean and interquartile ranges. Thin grey lines denote individual cells. Paired t-test. **c** Same as in b but for spatial information. **d** Correlations of spatial activity profiles ('place fields') before (*left, blue*) and after (*right, orange*) suppression of CA1 PV-IN activity *via* h4MDi. Dots denote individual cells. **e** Activity maps of CA1 place cells shown with the same sorting on the familiar (*left plot of group*) and novel (*right plot*) track. Data are shown separately for cells with familiar-context place fields during control conditions (*left group*, *blue shading*) and after clozapine injection (*right group, orange shading*). **f-j** Same as in a-e but for SOM-Cre mice in which activity of CA1 SOM-INs was suppressed via h4MDi. **k** Ratios of place cell transient rates after clozapine application divided by those under baseline conditions for familiar context (*brown*) and novel context (*yellow*) place cells in the DG (*left group*) and CA1 (*right group*) recorded in PV-Cre and SOM-Cre mice. See the corresponding section of **Supplementary Table 2** for 3-way ANOVA testing for interactions between IN type, hippocampal region, and novelty. **l** Same as in k but showing ratios of spatial information after clozapine application divided by baseline values. c,g,h Wilcoxon signed-rank sum test, d,i Wilcoxon rank-sum test, k,l Kruskal-Wallis ANOVA on Ranks with Dunn's post-hoc test. See the corresponding section of **Supplementary Table 2** for 3-way ANOVA testing for interactions between IN type, hippocampal region, and novelty; NS not significant; *$p < 0.05$; **$p < 0.01$, ***$p < 0.001$. For exact p values see **Supplementary Table 2**. Thick lines on violin plots indicate median and interquartile ranges. Thin lines and dots denote values for individual cells.

Supplementary Table 1 | Mice, experiments, and observation numbers

Mouse ID	Strain	Virus	Data shown on Figures	Unit	Region (N sessions)	Region (N units)
142	PV-Cre	AAV1.CAG.FLEX.mRuby2. GSG.P2A.GCaMP6f.WPRE.pA	Figs.1D-K, 2A-D, 3A-E, 4A,B<br><br>Supplementary Figs. 1A-I, 2A-F, 3A-H, 4A-G, 5A-F	Interneurons	CA1 (2)	CA1 (115)
143					CA1 (2)	CA1 (95)
144					CA1 (1)	CA1 (38)
2388					CA1 (1)	CA1 (7)
2389					CA1 (3)	CA1 (20)
2797					CA1 (1), DG (3)	CA1 (9), DG (33)
2815					CA1 (1), DG (1)	CA1 (5), DG (5)
2816					CA1 (1)	CA1 (17)
11					CA3 (1), DG (1)	CA3 (67), DG (15)
12					CA3 (1)	CA3 (6)
13					CA3 (1)	CA3 (11)
18					CA3 (2), DG (1)	CA3 (25), DG (3)
19					DG (2)	DG (20)
20					DG (2)	DG (42)
3202					DG (1)	DG (15)
1376	SOM-Cre				CA1 (1)	CA1 (10)
1523					CA1 (2)	CA1 (20)
1525					CA1 (2)	CA1 (13)
1541					CA1 (1)	CA1 (10)
1627					CA1 (1)	CA1 (13)
1719					CA1 (2)	CA1 (34)
1724					CA1 (1)	CA1 (13)
197					CA3 (2)	CA3 (136)
1891					CA3 (1)	CA3 (9)
1892					CA3 (2)	CA3 (62)
1905					CA3 (1)	CA3 (5)
1909					CA3 (2)	CA3 (25)
1524					DG (1)	DG (16)
1628					DG (2)	DG (22)
1629					DG (1)	DG (12)
1630					DG (1)	DG (18)
1718					DG (3)	DG (51)
1077	SOM-Cre	AAV9.hSyn.flex.GAP43-GCaMP6s	Fig. 4C,D<br><br>Supplementary Fig. 6A-G	Axons	Hilus (2), ML (3)	Hilus (673), ML (258)
1079					Hilus (3), ML (3)	Hilus (1019), ML (455)
1081					Hilus (3), ML (2)	Hilus (765), ML (244)
1083					ML (3)	ML (239)
373	PV-Cre	AAV2-hSyn-DIO-hM4D(Gi)-mCherry + AAV1-Syn-Gcamp6f	Figs. 5A-D, 6A-H,L,M, 6A-D<br><br>Supplementary Figs. 2G-P, 7A-D, 8K,L	Granule cells	DG (2)	DG (106)
375					DG (1)	DG (53)
379					DG (1)	DG (89)
718					DG (3)	DG (408)
719					DG (6)	DG (793)
720					DG (2)	DG (164)
805	SOM-Cre				DG (4)	DG (796)
891					DG (1)	DG (54)
892					DG (3)	DG (376)
893					DG (4)	DG (235)
895					DG (3)	DG (307)
916					DG (2)	DG (136)
755	PV-Cre		Supplementary Fig. 8A-L	Pyramidal cells	CA1 (1)	CA1 (801)
757					CA1 (1)	CA1 (834)
899	SOM-Cre				CA1 (1)	CA1 (994)
903					CA1 (1)	CA1 (655)
219	PV-Cre	AAV2-hSyn-DIO-mCherry + AAV1-Syn-Gcamp6f (DREADD CONTROLS)	Figs. 6I-M, 7E,F	Granule Cells	DG (2)	DG (149)
626					DG (2)	DG (248)
635					DG (1)	DG (202)
1107	SOM-Cre				DG (3)	DG (165)
1109					DG (3)	DG (200)

Supplementary Table 2 | Tabulated summary of all statistics throughout the manuscript.

Notes:

a) *The reported n numbers exclude missing ('NaN') values.*
b) *All group comparisons are two-sided*
c) *'00e+00' and '1.00e+00' denote p-values that approach 0 or 1, respectively, with intervals smaller than machine precision.*

Figure	Unit of comparison	Variable	n	test	results
Fig 2B	Cell populations	Fraction of speed modulated	6 groups	Chi-square	Chi2 = 141.56; p = 2.015163e-25
Fig 2C	Cells	Speed tuning slope	CA1PV: 302<br>CA1SOM: 111<br>CA3PV: 109<br>CA3SOM: 235<br>DGPV: 121<br>DGSOM: 118	Kruskal-Wallis with Dunn's post hoc test	Kruskal-Wallis<br>Source SS df MS Chi-sq Prob>Chi-sq<br>Groups 1.06e+07 5 2.12e+06 127.86 6.77e-26<br>Error 7.18e+07 990 7.25e+04<br>Total 8.23e+07 995<br><br>Dunn's post-hoc test<br>Group1 Group2 p<br>CA1PV CA1SOM 1.00e+00<br>CA1PV CA3PV 6.75e-01<br>CA1PV CA3SOM 9.91e-03<br>CA1PV DGPV 2.47e-02<br>CA1PV DGSOM 8.33e-15<br>CA1SOM CA3PV 9.81e-01<br>CA1SOM CA3SOM 3.30e-01<br>CA1SOM DGPV 5.80e-02<br>CA1SOM DGSOM 5.33e-11<br>CA3PV CA3SOM 1.00e+00<br>CA3PV DGPV 6.67e-04<br>CA3PV DGSOM 6.66e-15<br>CA3SOM DGPV 2.15e-07<br>CA3SOM DGSOM 0.00e+00<br>DGPV DGSOM 4.35e-04
Fig 2D	Cells	Moving to Immobile ratio	CA1PV: 305<br>CA1SOM: 112<br>CA3PV: 109<br>CA3SOM: 237<br>DGPV: 133<br>DGSOM: 119	Kruskal-Wallis with Dunn's post hoc test	Kruskal-Wallis<br>Source SS df MS Chi-sq Prob>Chi-sq<br>Groups 1.13e+07 5 2.26e+06 131.39 1.21e-26<br>Error 7.58e+07 1009 7.52e+04<br>Total 8.71e+07 1014<br><br>Dunn's post-hoc test<br>Group1 Group2 p<br>CA1PV CA1SOM 6.07e-02<br>CA1PV CA3PV 8.90e-01<br>CA1PV CA3SOM 8.82e-01<br>CA1PV DGPV 2.60e-05<br>CA1PV DGSOM 0.00e+00<br>CA1SOM CA3PV 9.90e-01<br>CA1SOM CA3SOM 8.08e-01<br>CA1SOM DGPV 9.26e-01<br>CA1SOM DGSOM 1.32e-09<br>CA3PV CA3SOM 1.00e+00<br>CA3PV DGPV 1.46e-01<br>CA3PV DGSOM 5.31e-13<br>CA3SOM DGPV 1.06e-02<br>CA3SOM DGSOM 0.00e+00<br>DGPV DGSOM 1.41e-06
Fig 2D	Cells	Moving to Immobile ratio	CA1PV: 305<br>CA1SOM: 112<br>CA3PV: 109<br>CA3SOM: 237<br>DGPV: 133<br>DGSOM: 119	Signed rank-sum test (mobile vs. immobile activity)	Signed ranks p-value<br>CA1PV: 2.606022e-44<br>CA1SOM: 6.850084e-17<br>CA3PV: 7.748400e-12<br>CA3SOM: 1.552508e-25<br>DGPV: 2.882399e-16<br>DGSOM: 2.454414e-01
Fig 3B	Cell populations	Fraction of cells with significant SI	6 groups	Chi-square	Chi2 = 7.08; p = 2.147593e-01
Fig 3C	Cells	Spatial information (normalized)	CA1PV: 290<br>CA1SOM: 112<br>CA3PV: 108<br>CA3SOM: 230<br>DGPV: 130<br>DGSOM: 109	Kruskal-Wallis with Dunn's post hoc test	Kruskal-Wallis<br>Source SS df MS Chi-sq Prob>Chi-sq<br>Groups 7.09e+06 5 1.42e+06 88.68 1.27e-17<br>Error 7.11e+07 973 7.31e+04<br>Total 7.82e+07 978<br><br>Dunn's post-hoc test<br>Group1 Group2 p<br>CA1PV CA1SOM 8.93e-01<br>CA1PV CA3PV 2.83e-14<br>CA1PV CA3SOM 1.74e-08<br>CA1PV DGPV 6.29e-07<br>CA1PV DGSOM 6.02e-05<br>CA1SOM CA3PV 8.96e-07<br>CA1SOM CA3SOM 1.82e-02

					CA1SOM DGPV 1.97e-02<br>CA1SOM DGSOM 1.22e-01<br>CA3PV CA3SOM 3.14e-02<br>CA3PV DGPV 2.01e-01<br>CA3PV DGSOM 7.80e-02<br>CA3SOM DGPV 1.00e+00<br>CA3SOM DGSOM 1.00e+00<br>DGPV DGSOM 1.00e+00
Fig 3C	Cells	Spatial information (normalized)	CA1PV: 290<br>CA1SOM: 112<br>CA3PV: 108<br>CA3SOM: 230<br>DGPV: 130<br>DGSOM: 109	Signed rank-sum test (real vs. bootstrapping)	**Signed ranks p-value**<br>CA1PV: 7.821840e-05<br>CA1SOM: 2.462118e-05<br>CA3PV: 8.114760e-02<br>CA3SOM: 5.242331e-08<br>DGPV: 1.854587e-03<br>DGSOM: 1.522845e-01
Fig 3D	Cells	Spatial coherence	CA1PV: 306<br>CA1SOM: 111<br>CA3PV: 109<br>CA3SOM: 237<br>DGPV: 133<br>DGSOM: 119	Kruskal-Wallis with Dunn’s post hoc test	Kruskal-Wallis<br>Source SS df MS Chi-sq Prob>Chi-sq<br>Groups 9.27e+06 5 1.85e+06 107.90 1.14e-21<br>Error 7.79e+07 1009 7.72e+04<br>Total 8.71e+07 1014<br><br>Dunn's post-hoc test<br>Group1 Group2 p<br>CA1PV CA1SOM 6.82e-05<br>CA1PV CA3PV 3.43e-01<br>CA1PV CA3SOM 3.65e-03<br>CA1PV DGPV 7.24e-09<br>CA1PV DGSOM 1.00e+00<br>CA1SOM CA3PV 5.50e-01<br>CA1SOM CA3SOM 1.07e-11<br>CA1SOM DGPV 0.00e+00<br>CA1SOM DGSOM 1.03e-04<br>CA3PV CA3SOM 1.70e-05<br>CA3PV DGPV 7.50e-11<br>CA3PV DGSOM 1.72e-01<br>CA3SOM DGPV 3.53e-02<br>CA3SOM DGSOM 4.49e-01<br>DGPV DGSOM 1.31e-04
Fig 3D	Cells	Spatial coherence	CA1PV: 306<br>CA1SOM: 111<br>CA3PV: 109<br>CA3SOM: 237<br>DGPV: 133<br>DGSOM: 119	Signed rank-sum test (real vs. bootstrapping)	**Signed ranks p-value**<br>CA1PV: 5.628403e-16<br>CA1SOM: 2.358677e-11<br>CA3PV: 1.913533e-07<br>CA3SOM: 7.368111e-04<br>DGPV: 2.051906e-05<br>DGSOM: 5.933777e-07
Fig 3E	Cells	Within-session stability (1st to 2nd half map-correlation)	CA1PV: 306<br>CA1SOM: 113<br>CA3PV: 109<br>CA3SOM: 237<br>DGPV: 133<br>DGSOM: 119	Kruskal-Wallis with Dunn’s post hoc test	Kruskal-Wallis<br>Source SS df MS Chi-sq Prob>Chi-sq<br>Groups 3.28e+06 5 6.56e+05 38.01 3.76e-07<br>Error 8.44e+07 1011 8.35e+04<br>Total 8.77e+07 1016<br>Note: Intervals can be used for testing but are not simultaneous confidence intervals.<br><br>Dunn's post-hoc test<br>Group1 Group2 p<br>CA1PV CA1SOM 1.00e+00<br>CA1PV CA3PV 1.76e-02<br>CA1PV CA3SOM 3.18e-01<br>CA1PV DGPV 9.98e-01<br>CA1PV DGSOM 8.96e-03<br>CA1SOM CA3PV 4.09e-01<br>CA1SOM CA3SOM 2.34e-01<br>CA1SOM DGPV 1.00e+00<br>CA1SOM DGSOM 3.23e-01<br>CA3PV CA3SOM 2.40e-05<br>CA3PV DGPV 4.82e-01<br>CA3PV DGSOM 1.00e+00<br>CA3SOM DGPV 9.59e-02<br>CA3SOM DGSOM 7.62e-06<br>DGPV DGSOM 3.86e-01
Fig 3E	Cells	Within-session stability (1st to 2nd half map-correlation)	CA1PV: 306<br>CA1SOM: 113<br>CA3PV: 109<br>CA3SOM: 237<br>DGPV: 133<br>DGSOM: 119	Signed rank-sum test (real vs. bootstrapping)	Signed ranks p-value<br>CA1PV: 1.467995e-15<br>CA1SOM: 1.189710e-06<br>CA3PV: 5.639416e-12<br>CA3SOM: 2.119533e-05<br>DGPV: 2.470213e-06<br>DGSOM: 1.359888e-11
Fig4B	Cells	Mean activity (familiar vs. novel)	CA1PV: 302<br>CA1SOM: 112<br>CA3PV: 103<br>CA3SOM: 234<br>DGPV: 133<br>DGSOM: 119	Signed rank-sum test	Signed ranks p-value<br>CA1PV: 3.813728e-01<br>CA1SOM: 1.327267e-02<br>CA3PV: 3.204042e-05<br>CA3SOM: 2.233279e-05<br>DGPV: 1.065350e-13<br>DGSOM: 1.150003e-13

Fig 4C,D	Boutons	Mean activity (familiar vs. novel)	ML Axons: 671<br>hilar Axons: 1317	Signed rank-sum test	Signed ranks p-value<br>Hilar Axons: p = 5.18e-07<br>ML Axons: p = 1.561e-15
Fig 5D	Interneurons	Mean activity (baseline vs. clozapine)	DGSOM: 63	paired-sample t-test	paired-sample p-value<br>DGSOM: 4.887e-09
Fig 5D	Interneurons	Mean activity (baseline vs. clozapine)	DGPV: 55	paired-sample t-test	paired-sample p-value<br>DGPV: 0.001436
Fig 6B	Sessions	Fraction of place cells	15	Paired t-test	Familiar bsl vs. clz: p = 0.005583<br>Novel bsl vs. clz: p = 0.253944
Fig 6B	Sessions	Fraction of place cells	15	2-way ANOVA	Source / Sum Sq. / d / Mean Sq. / F / Prob>F<br>IsNov / 4.05e+00 / 1 / 4.05 / 0.41 / 0.5231<br>IsClz / 2.73e+01 / 1 / 27.27 / 2.8 / 0.101<br>IsNov*IsClz / 4.10e+00 / 1 / 4.10 / 0.42 / 0.5207<br>Error / 5.49e+02 / 56 / 9.81<br>Total / 5.85e+02 / 59
Fig 6B	Proportions	Fraction of place cells	2	Fisher's exact test	Familiar bsl vs. clz p = 0.004691<br>Novel bsl vs. clz, p = 0.282346
Fig 6C	GC place cells	Transient-rates	Fam 97<br>Nov 77	Paired t-test	Familiar bsl vs. clz, p = 0.01994<br>Novel bsl vs. clz, p = 0.5654
Fig 6D	GC place cells	Spatial information	Fam 92<br>Nov 74	Signed rank-sum test	Familiar bsl vs. clz, p = 0.004993<br>Novel bsl vs. clz, p = 0.04364
Fig 6F	Sessions	Fraction of place cells	17	Paired t-test	Familiar bsl vs. clz: p = 0.096416<br>Novel bsl vs. clz: p = 0.098845
Fig 6F	Sessions	Fraction of place cells	17	2-way ANOVA	Source / Sum Sq. / d.f. / Mean Sq. / F / Prob>F<br>IsNov / 2.51e+01 / 1 / 25.07 / 1.9 / 0.1719<br>IsClz / 2.71e+01 / 1 / 27.14 / 2.1 / 0.1555<br>IsNov*IsClz / 3.25e-03 / 1 / 0.00 / 0.00025 / 0.9875<br>Error / 8.41e+02 / 64 / 13.14<br>Total / 8.93e+02 / 67
Fig 6F	Proportions	Fraction of place cells	2	Fisher's exact test	Familiar bsl vs. clz p = 0.211992<br>Novel bsl vs. clz, p = 0.486475
Fig 6G	GC place cells	Transient rates	Fam 217<br>Nov 174	Paired t-test	Familiar bsl vs. clz, p = 0.1743<br>Novel bsl vs. clz, p = 0.04956
Fig 6H	GC place cells	Spatial information	Fam 201<br>Nov 171	Signed rank-sum test	Familiar bsl vs. clz, p = 0.1387<br>Novel bsl vs. clz, p = 0.2648
Fig 6I	GC place cells	Place field correlations (fam - nov)	Bsl 146<br>Clz 156	Wilcoxon rank-sum test	Bsl vs. clz, p = 0.628
Fig 6J	GC place cells	Transient rates	Fam: 158<br>Nov: 138	Paired t-test	Fam bsl vs. clz: p = 0.1199<br>Nov bsl vs. clz: p = 0.6058
Fig 6K	GC place cells	Spatial information	Bsl: 152<br>Clz: 130	Signed rank-sum test	Fam bsl vs. clz: p = 0.9267<br>Nov bsl vs. clz: p = 0.9416
Fig 6L (left)	GC place cells	Transient rates (ratio pre-post clz)	PV: 87<br>SOM: 203<br>Ctr: 148	Kruskal-Wallis with Dunn's post hoc test	Kruskal-Wallis<br>Source / SS / df / MS / Chi-sq / Prob>Chi-sq<br>Groups / 2.42e+05 / 2 / 1.21e+05 / 15.10 / 5.26e-04<br>Error / 6.76e+06 / 435 / 1.55e+04<br>Total / 7.00e+06 / 437<br><br>Dunn's post-hoc test<br>Group1 / Group2 / p<br>PV / SOM / 3.08e-04<br>PV / Ctr / 2.35e-02<br>SOM / Ctr / 4.86e-01

Fig 6L (right)	GC place cells	Transient rates (ratio pre-post clz)	PV: 73<br>SOM: 169<br>Ctr: 129	Kruskal-Wallis with Dunn's post hoc test	Source SS df MS Chi-sq Prob>Chi-sq<br>Groups 3.51e+04 2 1.76e+04 3.06 2.17e-01<br>Error 4.22e+06 368 1.15e+04<br>Total 4.26e+06 370
Fig 6K	GC place cells	Transient rates (ratio pre-post clz)	PV Fam 87<br>PV Nov 73<br>SOM Fam 203<br>SOM Nov 169	2-Way ANOVA with Dunn's post-hoc test	Source Sum Sq. d.f. Mean Sq. F Prob>F<br>Genotype 3.47e+00 1 3.47 2.6 0.105<br>Familiarity 2.66e+00 1 2.66 2 0.1556<br>Genotype*Fam 5.45e+00 1 5.45 4.1 0.04246<br>Error 6.95e+02 528 1.32<br>Total 7.05e+02 531<br><br>Dunn's post-hoc test<br>Group1 Group2 p<br>PV,Fam SOM,Fam 3.41e-02<br>PV,Fam PV,Nov 1.64e-01<br>PV,Fam SOM,Nov 1.26e-01<br>SOM,Fam PV,Nov 9.99e-01<br>SOM,Fam SOM,Nov 9.44e-01<br>PV,Nov SOM,Nov 9.93e-01
Fig 6M (left)	GC place cells	Spatial information (ratio pre-post clz)	PV: 86<br>SOM: 202<br>Ctr: 147	Kruskal-Wallis with Dunn's post hoc test	Kruskal-Wallis<br>Source SS df MS Chi-sq Prob>Chi-sq<br>Groups 2.27e+05 2 1.13e+05 14.34 7.68e-04<br>Error 6.63e+06 432 1.54e+04<br>Total 6.86e+06 434<br><br>Dunn's post-hoc test<br>Group1 Group2 p<br>PV SOM 1.08e-03<br>PV Ctr 2.66e-03<br>SOM Ctr 1.00e+00
Fig 6M (right)	GC place cells	Spatial information (ratio pre-post clz)	PV: 72<br>SOM: 162<br>Ctr: 124	Kruskal-Wallis with Dunn's post hoc test	Kruskal-Wallis<br>Source SS df MS Chi-sq Prob>Chi-sq<br>Groups 6.97e+04 2 3.49e+04 6.51 3.85e-02<br>Error 3.75e+06 355 1.06e+04<br>Total 3.82e+06 357<br><br>Dunn's post-hoc test<br>Group1 Group2 p<br>PV SOM 3.27e-02<br>PV Ctr 3.39e-01<br>SOM Ctr 5.90e-01
Fig 6M	GC place cells	Spatial information (ratio pre-post clz)	PV Fam 86<br>PV Nov 72<br>SOM Fam 202<br>SOM Nov 162	2-Way ANOVA with Dunn's post-hoc test	Source Sum Sq. d.f. Mean Sq. F Prob>F<br>Genotype 2.61e+01 1 26.12 7.4 0.006673<br>Familiarity 1.96e-01 1 0.20 0.056 0.8134<br>Genotype*Fam 2.01e+00 0 2.01 0.57 0.45<br>Error 1.82e+03 518 3.52<br>Total 1.85e+03 521<br><br>Dunn's post-hoc test<br>Group1 Group2 p<br>PV,Fam SOM,Fam 4.77e-02<br>PV,Fam PV,Nov 9.34e-01<br>PV,Fam SOM,Nov 1.46e-01<br>SOM,Fam PV,Nov 3.06e-01<br>SOM,Fam SOM,Nov 9.65e-01<br>PV,Nov SOM,Nov 5.44e-01
Fig 7B	GC place cells	Place-field correlations FN	Fam 83<br>Nov 58	Wilcoxon rank-sum test	Correlations pre vs. post clozapine: p = 0.01646
Fig 7D	GC place cells	Place-field correlations FN	Fam 178<br>Nov 198	Wilcoxon rank-sum test	Correlations pre vs. post clozapine: p = 0.03102
Supplementary Fig. 2A	Animals	Speed modulation slope	CA1PV: 8<br>CA1SOM: 7<br>CA3PV: 4<br>CA3SOM: 5<br>DGPV: 7<br>DGSOM: 5	Kruskal-Wallis	Kruskal-Wallis<br>Source SS df MS Chi-sq Prob>Chi-sq<br>Groups 1.13e+03 5 2.26e+02 10.19 7.01e-02<br>Error 2.75e+03 30 9.18e+01<br>Total 3.89e+03 35
Supplementary Fig. 2A	Recording sessions	Speed modulation slope	CA1PV: 12<br>CA1SOM: 10<br>CA3PV: 5<br>CA3SOM: 8<br>DGPV: 11<br>DGSOM: 8	Kruskal-Wallis with Dunn's post hoc test	Kruskal-Wallis<br>Source SS df MS Chi-sq Prob>Chi-sq<br>Groups 3.72e+03 5 7.45e+02 15.05 1.02e-02<br>Error 9.39e+03 48 1.96e+02<br>Total 1.31e+04 53<br><br>Dunn's post-hoc test<br>Group1 Group2 p<br>CA1PV CA1SOM 1.00e+00<br>CA1PV CA3PV 1.00e+00<br>CA1PV CA3SOM 9.99e-01<br>CA1PV DGPV 9.90e-01<br>CA1PV DGSOM 8.28e-02<br>CA1SOM CA3PV 1.00e+00<br>CA1SOM CA3SOM 1.00e+00<br>CA1SOM DGPV 9.24e-01

					CA1SOM DGSOM 4.31e-02<br>CA3PV CA3SOM 1.00e+00<br>CA3PV DGPV 9.91e-01<br>CA3PV DGSOM 1.98e-01<br>CA3SOM DGPV 5.90e-01<br>CA3SOM DGSOM 1.19e-02<br>DGPV DGSOM 7.46e-01
Supplementary Fig. 2B	Animals	Activity ratio mobile - immobile	CA1PV: 8<br>CA1SOM: 7<br>CA3PV: 4<br>CA3SOM: 5<br>DGPV: 7<br>DGSOM: 5	Kruskal-Wallis	Kruskal-Wallis<br>Source SS df MS Chi-sq Prob>Chi-sq<br>Groups 1.08e+03 5 2.17e+02 9.77 8.20e-02<br>Error 2.80e+03 30 9.33e+01<br>Total 3.89e+03 35
Supplementary Fig. 2B	Recording sessions	Activity ratio mobile - immobile	CA1PV: 12<br>CA1SOM: 10<br>CA3PV: 5<br>CA3SOM: 8<br>DGPV: 11<br>DGSOM: 8	Kruskal-Wallis with Dunn's post hoc test	Kruskal-Wallis<br>Source SS df MS Chi-sq Prob>Chi-sq<br>Groups 4.37e+03 5 8.74e+02 17.66 3.40e-03<br>Error 8.75e+03 48 1.82e+02<br>Total 1.31e+04 53<br>Note: Intervals can be used for testing but are not simultaneous confidence intervals.<br><br>Dunn's post-hoc test<br>Group1 Group2 p<br>CA1PV CA1SOM 9.96e-01<br>CA1PV CA3PV 6.81e-01<br>CA1PV CA3SOM 4.29e-01<br>CA1PV DGPV 1.25e-01<br>CA1PV DGSOM 1.67e-03<br>CA1SOM CA3PV 9.98e-01<br>CA1SOM CA3SOM 9.92e-01<br>CA1SOM DGPV 8.86e-01<br>CA1SOM DGSOM 7.53e-02<br>CA3PV CA3SOM 1.00e+00<br>CA3PV DGPV 1.00e+00<br>CA3PV DGSOM 9.20e-01<br>CA3SOM DGPV 1.00e+00<br>CA3SOM DGSOM 8.11e-01<br>DGPV DGSOM 9.10e-01
Supplementary Fig. 2C	Animals	Spatial Information (normalized)	CA1PV: 8<br>CA1SOM: 7<br>CA3PV: 4<br>CA3SOM: 5<br>DGPV: 7<br>DGSOM: 5	Paired t-test	CA1-PV data vs. shuffle: p = 0.01632<br>CA1-SOM data vs. shuffle: p = 0.001761<br>CA2/3-PV data vs. shuffle: p = 0.09146<br>CA2/3-SOM data vs. shuffle: p = 0.6908<br>DG-PV data vs. shuffle: p = 0.04745<br>DG-SOM data vs. shuffle: p = 0.08355
Supplementary Fig. 2C	Recording sessions	Spatial Information (normalized)	CA1PV: 12<br>CA1SOM: 10<br>CA3PV: 5<br>CA3SOM: 8<br>DGPV: 11<br>DGSOM: 8	Paired t-test	CA1-PV data vs. shuffle: p = 0.02366<br>CA1-SOM data vs. shuffle: p = 0.0001046<br>CA2/3-PV data vs. shuffle: p = 0.1326<br>CA2/3-SOM data vs. shuffle: p = 0.84<br>DG-PV data vs. shuffle: p = 0.01106<br>DG-SOM data vs. shuffle: p = 0.05467
Supplementary Fig. 2D	Animals	Spatial Coherence	CA1PV: 8<br>CA1SOM: 7<br>CA3PV: 4<br>CA3SOM: 5<br>DGPV: 7<br>DGSOM: 5	Paired t-test	CA1-PV data vs. shuffle: p = 0.01632<br>CA1-SOM data vs. shuffle: p = 0.001761<br>CA2/3-PV data vs. shuffle: p = 0.09146<br>CA2/3-SOM data vs. shuffle: p = 0.6908<br>DG-PV data vs. shuffle: p = 0.04745<br>DG-SOM data vs. shuffle: p = 0.08355
Supplementary Fig. 2D	Recording sessions	Spatial Coherence	CA1PV: 12<br>CA1SOM: 10<br>CA3PV: 5<br>CA3SOM: 8<br>DGPV: 11<br>DGSOM: 8	Paired t-test	CA1-PV data vs. shuffle: p = 0.02366<br>CA1-SOM data vs. shuffle: p = 0.0001046<br>CA2/3-PV data vs. shuffle: p = 0.1326<br>CA2/3-SOM data vs. shuffle: p = 0.84<br>DG-PV data vs. shuffle: p = 0.01106<br>DG-SOM data vs. shuffle: p = 0.05467
Supplementary Fig. 2E	Animals	Within-session stability	CA1PV: 8<br>CA1SOM: 7<br>CA3PV: 4<br>CA3SOM: 5<br>DGPV: 7<br>DGSOM: 5	Paired t-test	CA1-PV data vs. shuffle: p = 0.002428<br>CA1-SOM data vs. shuffle: p = 0.02749<br>CA2/3-PV data vs. shuffle: p = 0.06814<br>CA2/3-SOM data vs. shuffle: p = 0.4354<br>DG-PV data vs. shuffle: p = 0.03594<br>DG-SOM data vs. shuffle: p = 0.008802
Supplementary Fig. 2E	Recording sessions	Within-session stability	CA1PV: 12<br>CA1SOM: 10<br>CA3PV: 5<br>CA3SOM: 8<br>DGPV: 11<br>DGSOM: 8	Paired t-test	CA1-PV data vs. shuffle: p = 0.0004692<br>CA1-SOM data vs. shuffle: p = 0.009178<br>CA2/3-PV data vs. shuffle: p = 0.0553<br>CA2/3-SOM data vs. shuffle: p = 0.6147<br>DG-PV data vs. shuffle: p = 0.008767<br>DG-SOM data vs. shuffle: p = 0.0009816
Supplementary Fig. 2F	Animals	Familiar vs. Novel context activity	CA1PV: 8<br>CA1SOM: 7<br>CA3PV: 4<br>CA3SOM: 5<br>DGPV: 7<br>DGSOM: 5	Paired t-test	CA1-PV data vs. shuffle: p = 0.4351<br>CA1-SOM data vs. shuffle: p = 0.8317<br>CA2/3-PV data vs. shuffle: p = 0.09165<br>CA2/3-SOM data vs. shuffle: p = 0.01851<br>DG-PV data vs. shuffle: p = 0.01359<br>DG-SOM data vs. shuffle: p = 0.06071

Supplementary Fig. 2F	Recording sessions	Familiar vs. Novel context activity	CA1PV: 12<br>CA1SOM: 10<br>CA3PV: 5<br>CA3SOM: 8<br>DGPV: 11<br>DGSOM: 8	Paired t-test	CA1-PV data vs. shuffle: p = 0.1897<br>CA1-SOM data vs. shuffle: p = 0.8326<br>CA2/3-PV data vs. shuffle: p = 0.1063<br>CA2/3-SOM data vs. shuffle: p = 0.02603<br>DG-PV data vs. shuffle: p = 0.001869<br>DG-SOM data vs. shuffle: p = 0.01618
Supplementary Fig. 2G-J	Animals (PV-Cre h4MDi)	Transient Rates, Fam, Nov; Spatial info Fam, Nov	5	Paired t-test	Transient Rate fam data control vs. cloz: p = 0.09418<br>Transient Rate nov data control vs. cloz: p = 0.9869<br>Spatial Info fam data control vs. cloz: p = 0.06676<br>Spatial Info nov data control vs. cloz: p = 0.04563
Supplementary Fig. 2G-J	Recording sessions (PV-Cre h4MDi)	Transient Rates, Fam, Nov; Spatial info Fam, Nov	13	Paired t-test	Transient Rate fam data control vs. cloz: p = 0.004314<br>Transient Rate nov data control vs. cloz: p = 0.6036<br>Spatial Info fam data control vs. cloz: p = 0.06264<br>Spatial Info nov data control vs. cloz: p = 0.03497
Supplementary Fig. 2K	Animals (PV-Cre h4MDi)	Fan-Nov place field correlation	5	Paired t-test	Bsl vs. clz: p = 0.04147
Supplementary Fig. 2K	Recording sessions (PV-Cre h4MDi)	Fan-Nov place field correlation	13	Paired t-test	Bsl vs. clz: p = 0.03827
Supplementary Fig. 2L-0	Animals (SOM-Cre h4MDi)	Transient Rates, Fam, Nov; Spatial info Fam, Nov	6	Paired t-test	Transient Rate (Hz) fam control vs. cloz: p = 0.07366<br>Transient Rate (Hz) nov data control vs. cloz: p = 0.3712<br>Spatial Info (bits/sec) fam data control vs. cloz: p = 0.02832<br>Spatial Info (bits/sec) nov data control vs. cloz: p = 0.3523
Supplementary Fig. 2L-O	Recording sessions (SOM-Cre h4MDi)	Transient Rates, Fam, Nov; Spatial info Fam, Nov	17	Paired t-test	Transient Rate (Hz) fam data control vs. cloz: p = 0.1012<br>Transient Rate (Hz) nov data control vs. cloz: p = 0.08744<br>Spatial Info (bits/sec) fam data control vs. cloz: p = 0.16<br>Spatial Info (bits/sec) nov data control vs. cloz: p = 0.2085
Supplementary Fig. 2P	Animals (SOM-Cre h4MDi)	Fan-Nov place field correlation	6	Paired t-test	Bsl vs. clz: p = 0.07105
Supplementary Fig. 2P	Recording sessions (SOM-Cre h4MDi)	Fan-Nov place field correlation	17	Paired t-test	Bsl. Vs. clz: p = 0.0076
Supplementary Fig. 3B	Sessions	Mean Running speed (SOM vs PV)	SOM: 26<br>PV: 28	t-test	SOM vs. PV: p = 0.5732
Supplementary Fig. 3C	Sessions	Number of laps completed (SOM vs PV)	SOM: 26<br>PV: 28	Wilcoxon Rank-sum	SOM vs. PV: p = 0.2987
Supplementary Fig. 3E	Sessions	Mean running speed per trial (5 Fam, 5 Nov)	54	Kruskal-Wallis	Kruskal-Wallis<br>Source SS df MS Chi-sq Prob>Chi-sq<br>Columns 1.82e+05 9 2.02e+04 7.48 5.87e-01<br>Error 1.29e+07 530 2.44e+04<br>Total 1.31e+07 539
Supplementary Fig. 3E	Sessions	Mean running speed per trial (5 Fam, 5 Nov)	54	Pearson' correlation coefficient (trial number vs. mean running speed)	R = -0.0613<br>P = 0.1549
Supplementary Fig. 3G	Sessions	Mean running speed (fam vs. nov)	54	Paired t-test	Fam vs. nov p = 0.4261
Supplementary Fig. 3H	Cells	Activity difference (novel – familiar)	CA1-PV: 302<br>CA1-SOM: 112<br>CA2/3-PV: 103<br>CA2/3-SOM: 234<br>DG-PV: 133<br>DG-SOM: 119	Kruskal-Wallis with Dunn's post hoc test	Kruskal-Wallis<br>Source SS df MS Chi-sq Prob>Chi-sq<br>Groups 1.20e+07 5 2.39e+06 142.42 5.48e-29<br>Error 7.21e+07 997 7.24e+04<br>Total 8.41e+07 1002<br>Note: Intervals can be used for testing but are not simultaneous confidence intervals.<br><br>Dunn's post-hoc test<br>Group1 Group2 p<br>CA1-PV CA1-SOM 7.97e-01<br>CA1-PV CA2/3-PV 1.06e-03<br>CA1-PV CA2/3-SOM 9.92e-02<br>CA1-PV DG-PV 3.71e-12<br>CA1-PV DG-SOM 4.61e-08<br>CA1-SOM CA2/3-PV 5.10e-01<br>CA1-SOM CA2/3-SOM 1.00e+00<br>CA1-SOM DG-PV 9.08e-05<br>CA1-SOM DG-SOM 6.06e-09<br>CA2/3-PV CA2/3-SOM 6.35e-01<br>CA2/3-PV DG-PV 2.47e-01

					CA2/3-PV DG-SOM 6.66e-15<br>CA2/3-SOM DG-PV 1.85e-05<br>CA2/3-SOM DG-SOM 1.03e-13<br>DG-PV DG-SOM 0.00e+00
Supplementary Fig. 4B	Cells	Tuning-vector length	CA1PV: 305<br>CA1SOM: 113<br>CA3PV: 108<br>CA3SOM: 233<br>DGPV: 126<br>DGSOM: 118	Signed-rank test against bootstrap	Signed ranks p-value<br>CA1PV: 1.203935e-09<br>CA1SOM: 1.200355e-04<br>CA3PV: 2.458846e-03<br>CA3SOM: 1.942979e-05<br>DGPV: 3.690581e-03<br>DGSOM: 1.229251e-11
Supplementary Fig. 4C	Cells	Positional information	CA1PV: 305<br>CA1SOM: 113<br>CA3PV: 109<br>CA3SOM: 237<br>DGPV: 133<br>DGSOM: 109	Signed-rank test against bootstrap	Signed ranks p-value<br>CA1PV: 5.598537e-04<br>CA1SOM: 5.305824e-07<br>CA3PV: 8.522812e-09<br>CA3SOM: 6.277273e-15<br>DGPV: 5.348455e-10<br>DGSOM: 2.996878e-10
Supplementary Fig. 4D	Cells	Spatial information (normalized)	CA1PYR: 1973<br>CA1PV: 295<br>CA1SOM: 112<br>CA3PV: 109<br>CA3SOM: 232<br>DGPV: 132<br>DGSOM: 112	Kruskal-Wallis with Dunn's post hoc test	Kruskal-Wallis<br>Source SS df MS Chi-sq Prob>Chi-sq<br>Groups 3.11e+08 6 5.18e+07 423.82 2.11e-88<br>Error 1.86e+09 2958 6.29e+05<br>Total 2.17e+09 2964<br><br>Dunn's post-hoc test<br>Group1 Group2 p<br>CA1 CA1PV 7.35e-11<br>CA1 CA1SOM 6.76e-08<br>CA1 CA3PV 0.00e+00<br>CA1 CA3SOM 0.00e+00<br>CA1 DGPV 0.00e+00<br>CA1 DGSOM 0.00e+00<br>CA1PV CA1SOM 9.92e-01<br>CA1PV CA3PV 7.60e-10<br>CA1PV CA3SOM 6.69e-06<br>CA1PV DGPV 2.42e-04<br>CA1PV DGSOM 7.75e-03<br>CA1SOM CA3PV 1.66e-04<br>CA1SOM CA3SOM 1.46e-01<br>CA1SOM DGPV 2.43e-01<br>CA1SOM DGSOM 7.08e-01<br>CA3PV CA3SOM 2.16e-01<br>CA3PV DGPV 4.61e-01<br>CA3PV DGSOM 1.90e-01<br>CA3SOM DGPV 1.00e+00<br>CA3SOM DGSOM 1.00e+00<br>DGPV DGSOM 1.00e+00
Supplementary Fig. 4E	Cells	Tuning-vector length	CA1PYR: 1987<br>CA1PV: 305<br>CA1SOM: 113<br>CA3PV: 109<br>CA3SOM: 236<br>DGPV: 132<br>DGSOM: 119	Kruskal-Wallis with Dunn's post hoc test	Kruskal-Wallis<br>Source SS df MS Chi-sq Prob>Chi-sq<br>Groups 1.17e+09 6 1.95e+08 1561.48 0.00e+00<br>Error 1.08e+09 2994 3.61e+05<br>Total 2.25e+09 3000<br><br>Dunn's post-hoc test<br>Group1 Group2 p<br>CA1 CA1PV 0.00e+00<br>CA1 CA1SOM 0.00e+00<br>CA1 CA3PV 0.00e+00<br>CA1 CA3SOM 0.00e+00<br>CA1 DGPV 0.00e+00<br>CA1 DGSOM 0.00e+00<br>CA1PV CA1SOM 1.00e+00<br>CA1PV CA3PV 8.94e-01<br>CA1PV CA3SOM 4.00e-01<br>CA1PV DGPV 9.83e-01<br>CA1PV DGSOM 9.66e-01<br>CA1SOM CA3PV 1.00e+00<br>CA1SOM CA3SOM 1.00e+00<br>CA1SOM DGPV 1.00e+00<br>CA1SOM DGSOM 7.80e-01<br>CA3PV CA3SOM 1.00e+00<br>CA3PV DGPV 1.00e+00<br>CA3PV DGSOM 2.00e-01<br>CA3SOM DGPV 1.00e+00<br>CA3SOM DGSOM 3.61e-02<br>DGPV DGSOM 3.27e-01
Supplementary Fig. 4F	Cells	Positional information	CA1: 2114<br>CA1PV: 305<br>CA1SOM: 113<br>CA3PV: 109<br>CA3SOM: 237<br>DGPV: 133<br>DGSOM: 112	Kruskal-Wallis with Dunn's post hoc test	Kruskal-Wallis<br>Source SS df MS Chi-sq Prob>Chi-sq<br>Groups 5.31e+07 6 8.85e+06 65.35 3.67e-12<br>Error 2.49e+09 3116 7.98e+05<br>Total 2.54e+09 3122<br>Note: Intervals can be used for testing but are not simultaneous confidence intervals.<br><br>Dunn's post-hoc test<br>Group1 Group2 p<br>CA1 CA1PV 1.92e-10<br>CA1 CA1SOM 9.29e-01

					CA1 CA3PV 3.19e-02<br>CA1 CA3SOM 1.00e+00<br>CA1 DGPV 1.00e+00<br>CA1 DGSOM 1.00e+00<br>CA1PV CA1SOM 5.15e-06<br>CA1PV CA3PV 1.38e-09<br>CA1PV CA3SOM 6.99e-06<br>CA1PV DGPV 4.44e-03<br>CA1PV DGSOM 4.81e-04<br>CA1SOM CA3PV 9.96e-01<br>CA1SOM CA3SOM 9.99e-01<br>CA1SOM DGPV 9.68e-01<br>CA1SOM DGSOM 1.00e+00<br>CA3PV CA3SOM 2.44e-01<br>CA3PV DGPV 1.52e-01<br>CA3PV DGSOM 6.78e-01<br>CA3SOM DGPV 1.00e+00<br>CA3SOM DGSOM 1.00e+00<br>DGPV DGSOM 1.00e+00
Supplementary Fig. 4G	Cells	Trial-by-trial correlation	CA1PYR: 1906<br>CA1PV: 305<br>CA1SOM: 113<br>CA3PV: 108<br>CA3SOM: 235<br>DGPV: 133<br>DGSOM: 119	Kruskal-Wallis with Dunn's post hoc test	Kruskal-Wallis<br>Source SS df MS Chi-sq Prob>Chi-sq<br>Groups 1.43e+08 6 2.38e+07 200.63 1.39e-40<br>Error 1.93e+09 2912 6.63e+05<br>Total 2.07e+09 2918<br><br>Dunn's post-hoc test<br>Group1 Group2 p<br>CA1 CA1PV 3.19e-13<br>CA1 CA1SOM 3.19e-06<br>CA1 CA3PV 9.01e-10<br>CA1 CA3SOM 0.00e+00<br>CA1 DGPV 1.18e-09<br>CA1 DGSOM 1.35e-02<br>CA1PV CA1SOM 1.00e+00<br>CA1PV CA3PV 9.16e-01<br>CA1PV CA3SOM 6.03e-01<br>CA1PV DGPV 9.99e-01<br>CA1PV DGSOM 9.75e-01<br>CA1SOM CA3PV 9.99e-01<br>CA1SOM CA3SOM 9.94e-01<br>CA1SOM DGPV 1.00e+00<br>CA1SOM DGSOM 9.72e-01<br>CA3PV CA3SOM 1.00e+00<br>CA3PV DGPV 1.00e+00<br>CA3PV DGSOM 2.41e-01<br>CA3SOM DGPV 1.00e+00<br>CA3SOM DGSOM 7.34e-02<br>DGPV DGSOM 5.31e-01
Supplementary 5C	Cells	Within-session stability (correlation 1st to 2nd half)	CA1PYR: 1737<br>CA1PV: 306<br>CA1SOM: 113<br>CA3PV: 109<br>CA3SOM: 237<br>DGPV: 133<br>DGSOM: 119	Kruskal-Wallis with Dunn's post hoc test	Kruskal-Wallis<br>Source SS df MS Chi-sq Prob>Chi-sq<br>Groups 1.90e+08 6 3.16e+07 300.13 7.66e-62<br>Error 1.55e+09 2747 5.65e+05<br>Total 1.74e+09 2753<br><br>Dunn's post-hoc test<br>Group1 Group2 p<br>CA1 CA1PV 0.00e+00<br>CA1 CA1SOM 3.42e-09<br>CA1 CA3PV 5.94e-03<br>CA1 CA3SOM 0.00e+00<br>CA1 DGPV 1.05e-10<br>CA1 DGSOM 4.84e-03<br>CA1PV CA1SOM 1.00e+00<br>CA1PV CA3PV 4.09e-02<br>CA1PV CA3SOM 6.32e-01<br>CA1PV DGPV 1.00e+00<br>CA1PV DGSOM 2.15e-02<br>CA1SOM CA3PV 6.66e-01<br>CA1SOM CA3SOM 4.21e-01<br>CA1SOM DGPV 1.00e+00<br>CA1SOM DGSOM 5.62e-01<br>CA3PV CA3SOM 1.65e-04<br>CA3PV DGPV 5.93e-01<br>CA3PV DGSOM 1.00e+00<br>CA3SOM DGPV 3.30e-01<br>CA3SOM DGSOM 5.80e-05<br>DGPV DGSOM 4.83e-01
Supplementary 5F	Cells with significant SI	Within-session stability (correlation 1st to 2nd half)	CA1PYR: 600<br>CA1PV: 38<br>CA1SOM: 16<br>CA3PV: 9<br>CA3SOM: 16<br>DGPV: 17<br>DGSOM: 10	Kruskal-Wallis with Dunn's post hoc test	Kruskal-Wallis<br>Source SS df MS Chi-sq Prob>Chi-sq<br>Groups 3.37e+06 6 5.62e+05 81.09 2.13e-15<br>Error 2.60e+07 699 3.71e+04<br>Total 2.93e+07 705<br><br>Dunn's post-hoc test<br>Group1 Group2 p<br>CA1 CA1PV 7.70e-07<br>CA1 CA1SOM 1.35e-01<br>CA1 CA3PV 9.80e-01<br>CA1 CA3SOM 3.48e-05<br>CA1 DGPV 3.59e-05

					CA1 DGSOM 3.19e-01<br>CA1PV CA1SOM 1.00e+00<br>CA1PV CA3PV 9.94e-01<br>CA1PV CA3SOM 1.00e+00<br>CA1PV DGPV 1.00e+00<br>CA1PV DGSOM 1.00e+00<br>CA1SOM CA3PV 1.00e+00<br>CA1SOM CA3SOM 9.52e-01<br>CA1SOM DGPV 9.73e-01<br>CA1SOM DGSOM 1.00e+00<br>CA3PV CA3SOM 7.86e-01<br>CA3PV DGPV 8.36e-01<br>CA3PV DGSOM 1.00e+00<br>CA3SOM DGPV 1.00e+00<br>CA3SOM DGSOM 9.98e-01<br>DGPV DGSOM 9.99e-01
Supplementary Fig. 6G	Boutons	Activity ratio familiar / novel	Molecular Layer axons: 627<br>Hilar axons: 1317	Wilcoxon Rank-sum test	Activity ratios (nov/fam) Hilus vs. ML: p = 1.783e-16
Supplementary Fig. 7A	GC place cells	Transient rates	PV bsl 96<br>PV clz 71<br>SOM bsl 188<br>SOM clz 204	Paired t-test	PV Bsl, fam vs. nov, p = 0.001384<br>PV Clz fam vs. nov, p = 0.7819<br>SOM Bsl, fam vs. nov, p = 0.01527<br>SOM Clz fam vs. nov, p = 0.1633
Supplementary Fig. 7B	GC place cells	Spatial information	PV bsl 91<br>PV clz 66<br>SOM bsl 182<br>SOM clz 190	Signed rank-sum test	PV Bsl, fam vs. nov, p = 0.002806<br>PV Clz fam vs. nov, p = 0.6026<br>SOM Bsl, fam vs. nov, p = 0.007055<br>SOM Clz fam vs. nov, p = 0.02611
Supplementary Fig. 7C	GC place cells	Place-field correlations (familiar 1st-to-2nd-half vs. familiar-to-novel	PV bsl 60<br>PV clz 83<br>SOM bsl 126<br>SOM clz 178	Student's t-test	PV-Cre animals, FF vs. FN correlations, p = 0.0735<br>SOM-Cre animals, FF vs. FN correlations, p = 5.036e-06
Supplementary Fig. 7D	Sessions	Population vector correlations	PV: 5<br>SOM: 11	Paired t-test	PV: p = 0.7767<br>SOM: p = 0.004819
Supplementary Fig. 8B	CA1 place cells	Transient rates	Fam: 506<br>Nov: 565	Paired t-test	Familiar bsl vs. clz, p = 9.038e-09<br>Novel bsl vs. clz, p = 0.02718
Supplementary Fig. 8C	CA1 place cells	Spatial information	Fam: 489<br>Nov: 540	Signed rank-sum test	Familiar bsl vs. clz, p = 0.001777<br>Novel bsl vs. clz, p = 0.4096
Supplementary Fig. 8D	CA1 place cells	Place-field correlations fam-to-nov	Fam: 492<br>Nov: 551	Wilcoxon rank-sum test	Correlations pre vs. post clozapine: p = 0.01276
Supplementary Fig. 8G	CA1 place cells	Transient rates	Fam: 716<br>Nov: 687	Signed rank-sum test	Familiar bsl vs. clz, p = 1.449e-39<br>Novel bsl vs. clz, p = 4.686e-24
Supplementary Fig. 8H	CA1 place cells	Spatial information	Fam: 698<br>Nov: 664	Signed rank-sum test	Familiar bsl vs. clz, p = 0.01523<br>Novel bsl vs. clz, p = 0.0174
Supplementary Fig. 8I	CA1 place cells	Place-field correlations fam-to-nov	Fam: 588<br>Nov: 780	Wilcoxon rank-sum test	Correlations pre vs. post clozapine: p = 6.774e-06
Supplementary Fig. 8K	Place cells	Transient rates (ratio pre-post clz)	DG pv fam 97<br>DG pv nov 77<br>DG som fam 217<br>DG som nov 174<br>CA1 pv fam 716<br>CA1 pv nov 687<br>CA1 som fam 506<br>CA1 som nov 565	3-Way ANOVA with Dunn's post-hoc test	Source Sum Sq. d.f. Mean Sq. F Prob>F<br>SOM 4.12e+00 1 4.12 5.7 0.01742<br>Nov 5.02e-03 1 0.01 0.0069 0.9338<br>CA1 4.34e+00 1 4.34 6 0.01472<br>SOM*Nov 4.66e-01 1 0.47 0.64 0.424<br>SOM*CA1 2.99e+00 1 2.99 4.1 0.04292<br>Nov*CA1 2.77e+00 1 2.77 3.8 0.05109<br>Error 2.12e+03 2916 0.73<br>Total 2.14e+03 2922<br><br>Dunn's post-hoc test<br>Group1 Group2 p<br>PV,Fam,DG SOM,Fam,DG 1.70e-01<br>PV,Fam,DG PV,Nov,DG 9.37e-01<br>PV,Fam,DG SOM,Nov,DG 2.13e-01<br>PV,Fam,DG PV,Fam,CA1 1.69e-02<br>PV,Fam,DG SOM,Fam,CA1 6.58e-03<br>PV,Fam,DG PV,Nov,CA1 2.27e-01<br>PV,Fam,DG SOM,Nov,CA1 1.64e-01<br>SOM,Fam,DG PV,Nov,DG 9.65e-01<br>SOM,Fam,DG SOM,Nov,DG 9.98e-01<br>SOM,Fam,DG PV,Fam,CA1 9.92e-01<br>SOM,Fam,DG SOM,Fam,CA1 7.63e-01<br>SOM,Fam,DG PV,Nov,CA1 1.00e+00<br>SOM,Fam,DG SOM,Nov,CA1 1.00e+00

					PV,Nov,DG SOM,Nov,DG 5.44e-01<br>PV,Nov,DG PV,Fam,CA1 6.08e-01<br>PV,Nov,DG SOM,Fam,CA1 1.71e-01<br>PV,Nov,DG PV,Nov,CA1 8.70e-01<br>PV,Nov,DG SOM,Nov,CA1 9.45e-01<br>SOM,Nov,DG PV,Fam,CA1 1.00e+00<br>SOM,Nov,DG SOM,Fam,CA1 9.98e-01<br>SOM,Nov,DG PV,Nov,CA1 9.97e-01<br>SOM,Nov,DG SOM,Nov,CA1 9.83e-01<br>PV,Fam,CA1 SOM,Fam,CA1 9.90e-01<br>PV,Fam,CA1 PV,Nov,CA1 9.50e-01<br>PV,Fam,CA1 SOM,Nov,CA1 8.83e-01<br>SOM,Fam,CA1 PV,Nov,CA1 4.59e-01<br>SOM,Fam,CA1 SOM,Nov,CA1 2.04e-01<br>PV,Nov,CA1 SOM,Nov,CA1 1.00e+00
Supplementary Fig. 8L	Place cells	Spatial information (ratio pre-post clz)	DG PV Fam 92<br>DG PV Nov 74<br>DG SOM Fam 201<br>DG SOM Nov 171<br>CA1 pv fam 698<br>CA1 pv nov 687<br>CA1 som fam 489<br>CA1 soms nov 565	3-Way ANOVA with Dunn's post-hoc test	Source Sum Sq. d.f. Mean Sq. F Prob>F<br>IsSOM 3.52e-01 1 0.35 0.11 0.7367<br>IsNov 3.10e+00 1 3.10 1 0.3184<br>IsCA1 7.09e+00 1 7.09 2.3 0.1314<br>IsSOM*IsNov 3.32e+00 3.32 1.1 0.3022<br>IsSOM*IsCA1 7.91e+01 79.08 25 4.997e-07<br>IsNov*IsCA1 4.59e+00 1 4.59 1.5 0.225<br>Error 8.71e+03 2797 3.12<br>Total 8.87e+03 2803<br><br>Dunn's post-hoc test<br>Group1 Group2 p<br>PV,Fam,DG SOM,Fam,DG 3.41e-02<br>PV,Fam,DG PV,Nov,DG 1.00e+00<br>PV,Fam,DG SOM,Nov,DG 3.41e-01<br>PV,Fam,DG PV,Fam,CA1 9.85e-04<br>PV,Fam,DG SOM,Fam,CA1 5.56e-01<br>PV,Fam,DG PV,Nov,CA1 4.01e-02<br>PV,Fam,DG SOM,Nov,CA1 1.00e+00<br>SOM,Fam,DG PV,Nov,DG 4.29e-01<br>SOM,Fam,DG SOM,Nov,DG 1.00e+00<br>SOM,Fam,DG PV,Fam,CA1 9.80e-01<br>SOM,Fam,DG SOM,Fam,CA1 6.97e-01<br>SOM,Fam,DG PV,Nov,CA1 1.00e+00<br>SOM,Fam,DG SOM,Nov,CA1 1.22e-02<br>PV,Nov,DG SOM,Nov,DG 2.91e-01<br>PV,Nov,DG PV,Fam,CA1 4.36e-02<br>PV,Nov,DG SOM,Fam,CA1 8.26e-01<br>PV,Nov,DG PV,Nov,CA1 9.25e-02<br>PV,Nov,DG SOM,Nov,CA1 1.00e+00<br>SOM,Nov,DG PV,Fam,CA1 8.80e-01<br>SOM,Nov,DG SOM,Fam,CA1 9.54e-01<br>SOM,Nov,DG PV,Nov,CA1 1.00e+00<br>SOM,Nov,DG SOM,Nov,CA1 4.69e-02<br>PV,Fam,CA1 SOM,Fam,CA1 1.03e-02<br>PV,Fam,CA1 PV,Nov,CA1 9.53e-01<br>PV,Fam,CA1 SOM,Nov,CA1 2.95e-07<br>SOM,Fam,CA1 PV,Nov,CA1 2.76e-01<br>SOM,Fam,CA1 SOM,Nov,CA1 8.99e-02<br>PV,Nov,CA1 SOM,Nov,CA1 1.37e-05
