# Supplementary material for: Subfield-specific interneuron circuits govern the hippocampal response to novelty in male mice
Source: Nat Commun. 2024 Jan 24;15:714. doi: 10.1038/s41467-024-44882-3 (PMC10808551; doi:10.1038/s41467-024-44882-3)
Supplement: Supplementary file 3 — Reporting Summary [file 41467_2024_44882_MOESM3_ESM.pdf]

nature portfolio

Corresponding author(s): Dr. Thomas Hainmueller
Dr. Marlene Bartos

Last updated by author(s): Nov 23, 2023

Reporting Summary

Nature Portfolio wishes to improve the reproducibility of the work that we publish. This form provides structure for consistency and transparency in reporting. For further information on Nature Portfolio policies, see our Editorial Policies and the Editorial Policy Checklist.

Statistics

For all statistical analyses, confirm that the following items are present in the figure legend, table legend, main text, or Methods section.

n/a	Confirmed	
☐	☒	The exact sample size (n) for each experimental group/condition, given as a discrete number and unit of measurement
☐	☒	A statement on whether measurements were taken from distinct samples or whether the same sample was measured repeatedly
☐	☒	The statistical test(s) used AND whether they are one- or two-sided<br>*Only common tests should be described solely by name; describe more complex techniques in the Methods section.*
☐	☒	A description of all covariates tested
☐	☒	A description of any assumptions or corrections, such as tests of normality and adjustment for multiple comparisons
☐	☒	A full description of the statistical parameters including central tendency (e.g. means) or other basic estimates (e.g. regression coefficient) AND variation (e.g. standard deviation) or associated estimates of uncertainty (e.g. confidence intervals)
☐	☒	For null hypothesis testing, the test statistic (e.g. F, t, r) with confidence intervals, effect sizes, degrees of freedom and P value noted<br>*Give P values as exact values whenever suitable.*
☒	☐	For Bayesian analysis, information on the choice of priors and Markov chain Monte Carlo settings
☒	☐	For hierarchical and complex designs, identification of the appropriate level for tests and full reporting of outcomes
☐	☒	Estimates of effect sizes (e.g. Cohen's d, Pearson's r), indicating how they were calculated

Our web collection on statistics for biologists contains articles on many of the points above.

Software and code

Policy information about availability of computer code

Data collection	Matlab 2019b (MathWorks), ScanBox 2.0 (Neurolabware)
Data analysis	Matlab 2019b + custom code (https://github.com/ThomasHainmueller/HainmuellerCazala_et_al_2023), Suite2p 1.0 (github.com/MouseLand/suite2p)

For manuscripts utilizing custom algorithms or software that are central to the research but not yet described in published literature, software must be made available to editors and reviewers. We strongly encourage code deposition in a community repository (e.g. GitHub). See the Nature Portfolio guidelines for submitting code & software for further information.

Data

Policy information about availability of data

All manuscripts must include a data availability statement. This statement should provide the following information, where applicable:

- Accession codes, unique identifiers, or web links for publicly available datasets
- A description of any restrictions on data availability
- For clinical datasets or third party data, please ensure that the statement adheres to our policy

The processed data for all figures in this manuscript are available through the corresponding Source Data file. The raw dataset on which they are based is available from the corresponding authors upon request.

Research involving human participants, their data, or biological material

Policy information about studies with human participants or human data. See also policy information about sex, gender (identity/presentation), and sexual orientation and race, ethnicity and racism.

Reporting on sex and gender	N/a
Reporting on race, ethnicity, or other socially relevant groupings	N/a
Population characteristics	N/a
Recruitment	N/a
Ethics oversight	N/a

Note that full information on the approval of the study protocol must also be provided in the manuscript.

Field-specific reporting

Please select the one below that is the best fit for your research. If you are not sure, read the appropriate sections before making your selection.

☒ Life sciences ☐ Behavioural & social sciences ☐ Ecological, evolutionary & environmental sciences

For a reference copy of the document with all sections, see nature.com/documents/nr-reporting-summary-flat.pdf

Life sciences study design

All studies must disclose on these points even when the disclosure is negative.

Sample size	We recorded from several hundreds of neurons per group, as individually reported. No sample-size calculation was performed. Animal numbers were chosen to be in the range of comparable publications (e.g. Danielson et al., 2016, Neuron; Sheffield et al., 2017, Neuron; Hainmueller and Bartos, 2018, Nature), typically between 5-8 mice per group.
Data exclusions	Recording quality was assessed in preliminary recordings and only animals with appropriate optical access for imaging were included in experiments. All data sets that were recorded in experiments were included in the final manuscript.
Replication	The principal findings of the study were replicated in multiple cohorts of animals by different experimenters (22 animals were recorded by T.H., 10 animals were recorded by L.W.H., 25 animals were recorded by A.C.). Each individual finding was replicated in 2-8 animals (see Supplementary table 1, which contains numbers of animals tested in each individual experiment) and multiple independent repeats of the experiment were performed on repeated days for each animal, using different virtual environments (see Supplementary Table 1 for numbers of experiments in each animal).
Randomization	The study describes neuronal activity related to external variables in animals. Each animal serves as their own control in pre-post comparisons of various conditions. There were no pre-determined assignments to experimental groups in which randomization of subjects is relevant.
Blinding	Given the nature of experiments as outlined above, blinding of experimenters to the experimental conditions was not possible (e.g. it was obvious whether the animal is in a familiar or novel context or whether the animal is a PV-Cre or Som-Cre mouse). Given that neuronal activity was recorded continuously throughout the entire experiment and all data acquisition and processing steps would equally apply to all conditions compared in our analysis, there is further no possible benefit of blinding experimenters.

Reporting for specific materials, systems and methods

We require information from authors about some types of materials, experimental systems and methods used in many studies. Here, indicate whether each material, system or method listed is relevant to your study. If you are not sure if a list item applies to your research, read the appropriate section before selecting a response.

Materials & experimental systems

n/a	Involved in the study
☐	☒ Antibodies
☒	☐ Eukaryotic cell lines
☒	☐ Palaeontology and archaeology
☐	☒ Animals and other organisms
☒	☐ Clinical data
☒	☐ Dual use research of concern
☒	☐ Plants

Methods

n/a	Involved in the study
☒	☐ ChIP-seq
☒	☐ Flow cytometry
☒	☐ MRI-based neuroimaging

Antibodies

Antibodies used	primary anti parvalbumin (polyclonal rabbit, 1:1000, Swant; catalog no.: PV27, LOT 2014; validation in PV-KO mice), primary anti somatostatin (polyclonal rabbit, 1:500, Peninsula Laboratories; catalog no.: T4102, LOT A18PO21141; validation with Elisa and IHC); goat-anti-rabbit coupled to Alexa-Fluor 647 (1:1000, Abcam; catalog no.: AB150079, LOT GR3444080-3; validated by immunohistochemistry).
Validation	Swant PV27 was tested on brain slices and brain extract of C57/BL6 and parvalbumin knockout mice demonstrating specificity as per the suppliers material datasheet (https://www.swant.com/pdfs/PV27_Rabbit_anti_Parvalbumin.pdf). Penninsula rabbit anti-Somatostatin-14 was tested by Elisa and immunohistochemistry per supplier datasheet (https://www.bma.ch/files/product/T-4102.pdf). Both antibodies were found to replicate well known staining patterns expected in hippocampal tissue and staining co-localizes with Cre-driven transgenic labelling in the respective mouse lines in our experiments. goat-anti-rabbit coupled to Alexa-Fluor 647 (1:1000, Abcam; catalog no.: AB150079, LOT GR3444080-3 was validated by immunohistochemistry).

Animals and other research organisms

Policy information about studies involving animals; ARRIVE guidelines recommended for reporting animal research, and Sex and Gender in Research

Laboratory animals	We used a total of 26 PV-Cre (B6;129P2-Pvalbtm1(Cre)Arbr/J; The Jackson laboratory) and 31 SOM-IRES-Cre (Ssttm2.1(Cre)Zjh/J; The Jackson Laboratory) male mice aged 9-12 postnatal weeks at the beginning of experiments. In addition to the above, data from two B6;129P2-Pvalbtm1(cre)Arbr/J mice (PV-Cre; The Jackson laboratory) crossed with B6.Cg-Gt(ROSA)26Sortm9(CAG-tdTomato)Hze/J mice (Ai9-reporter; The Jackson laboratory) male mice aged 9-12 postnatal weeks at the beginning of experiments were included for the comparison of interneuron activity with principal cell activity in (Supplementary Figs. 4,5).
Wild animals	No wild animals were used in this study.
Reporting on sex	Male mice were used in this study to reduce variance between groups. Their larger body size and muscle mass facilitates carrying of surgical implant as well as performance in the behavioral head-fixed apparatus. Sex was assigned by genital morphological features.
Field-collected samples	No field-collected samples were used in this study.
Ethics oversight	All experiments involving animals were carried out according to national and institutional guidelines and approved by the 'Tierversuchskommission' of the Regierungspräsidium Freiburg (license no. G16/037) in accordance with German national legislation.

Note that full information on the approval of the study protocol must also be provided in the manuscript.

Plants

Seed stocks	No plants were used in this study.
Novel plant genotypes	No plants were used in this study.
Authentication	No plants were used in this study.
